# Exploiting social graph networks for emotion prediction

**DOI:** 10.1038/s41598-023-32825-9

**Published:** 2023-04-13

**Authors:** Maryam Khalid, Akane Sano

**Affiliations:** Computational Wellbeing Group, Department of Electrical and Computer Engineering, Rice University, 6500 Main Street, Houston, 77005 TX USA

**Keywords:** Health care, Electrical and electronic engineering, Computational science, Computer science

## Abstract

Emotion prediction plays an essential role in mental healthcare and emotion-aware computing. The complex nature of emotion resulting from its dependency on a person’s physiological health, mental state, and his surroundings makes its prediction a challenging task. In this work, we utilize mobile sensing data to predict self-reported happiness and stress levels. In addition to a person’s physiology, we also incorporate the environment’s impact through weather and social network. To this end, we leverage phone data to construct social networks and develop a machine learning architecture that aggregates information from multiple users of the graph network and integrates it with the temporal dynamics of data to predict emotion for all users. The construction of social networks does not incur additional costs in terms of ecological momentary assessments or data collection from users and does not raise privacy concerns. We propose an architecture that automates the integration of the user’s social network in affect prediction and is capable of dealing with the dynamic distribution of real-life social networks, making it scalable to large-scale networks. The extensive evaluation highlights the prediction performance improvement provided by the integration of social networks. We further investigate the impact of graph topology on the model’s performance.

## Introduction

Predicting emotion from passive sources can help regulate mental health, prevent breakdown/suicide, and make machines affect-intelligent. Mental health problems are impacting millions of people throughout the world with suicide being the third leading cause of death among young people^1^. Mental illnesses not only impact an individual’s work performance^2^ but also compromise the quality of life and relationships^3^. Emotion dysregulation is closely related to multiple mental health illnesses^4^ that can be managed if emotions are tracked, and intervention is provided in time. Multiple works^5–8^ provide evidence for the efficacy of emotion-regulating interventions in preventing critical mental health conditions. If predictions for mood and stress levels are available, they can be utilized to suggest interventions and regulate the behavior before a person reaches a high-stress or depressed condition. The work can be easily extended to other emotions such as fear, anger, or other health-related conditions. Emotion and well-being prediction using ubiquitous sensors and computing cannot only help humans improve their mental health^9^ but also make the machines more affect-intelligent. Machines and applications that incorporate user emotion in their operation can significantly improve the user experience.

Emotion is a complex entity resulting from a human’s current mental condition (internal dynamics) and external factors such as weather and social interactions. This elaborate nature of emotion makes its prediction a challenging task. There is no device to directly measure emotion; however, there are several passive data sources that are indicative of a person’s emotional state. Given the complex nature of emotion, one modality is not sufficient and data from multiple modalities need to be fused to obtain an accurate prediction. Another important aspect of emotion is that an emotional state is caused by several temporally-correlated factors over time. Instantaneous measurements of modalities in most cases are not able to predict an emotional state with high accuracy. On the other hand, when viewed as a sequence, trends start to appear. Some data sources that are widely discussed in the literature include video, speech, and, wearable data^10–12^, etc. Additional sources that are very easy to collect at high frequency without intrusion are physiological signals, weather, and information about the environment.

One important aspect of the environment is people. Previous studies show that people can transfer emotions to other people around them and this phenomenon is known as emotion contagion. Formally, emotion contagion is defined as the “phenomenon where the observed behavior of one individual leads to the reflexive production of the same behavior by others”^13,14^. This can happen through face-to-face interaction, voice, text, or movements^15^ in an individual or group setting over time spans varying from seconds to weeks^16^. There is evidence for contagious behavior in the spread of health and wellbeing indicators such as pneumonia^17^, obesity^18^, severe acute respiratory syndrome^19^, and sleep^20^. The network analysis in^17^ and^19^ illustrate how diseases spread in a network. Interesting findings in^18,20^ indicate that well-being behaviors such as sleep and obesity have discernable clusters. Furthermore, a person has a higher probability of exhibiting an attribute if their friend also possesses it e.g. obesity. Expanding on previous evidence suggesting the spread of emotion in a network,^21^ investigates whether happiness spreads from one person to another by leveraging graph networks. They utilize data collected over 20 years in Framingham Heart Study with 5124 participants and leverage the relationship and friendship information to construct graphs. After conducting a regression statistical analysis on these graphs, distinct clusters of happy and unhappy people are observed. Furthermore, their analysis showed that indirect ties till the depth of three and centrality in the network also impact future happiness. These works provide empirical evidence for the existence of emotion contagion but do not leverage it to predict emotion. Motivated by these findings, we develop a machine-learning solution that incorporates contagion while predicting future emotion levels.

Multiple deep learning models have been developed to predict self-reported mood or stress scores using multimodal data such as physiological, behavioral, and social interaction data, many of which are summarized in^22^. However, they do not account for user-to-user social interactions. The work in^12,23,24^ predicts the next day’s stress and happiness score from weather, physiological and behavioral data and accounts for the individual differences between users using personalized multi-task learning neural network models, gaussian process for domain adaptation, or fine-tuning neural network models such as convolutional neural network (CNN) and/or long-short term neural network (LSTM) to aggregate spatial and temporal aggregation of multimodal data. In addition to physiological data, these works also consider social interaction features such as the number of calls and SMS made during a day. However, these models do not combine information from multiple users to predict users’ mood or stress scores. In this work, we account for the impact other people would have on one’s mood and stress by integrating two aspects in the prediction model, (1) the user’s own multi-modal data and (2) multi-sensor data from other people that the user interacted with. To this end, we construct a prediction model that is complemented by an additional graph structure allowing the model to aggregate the information from multiple users based on their social interactions.

There are multiple existing works that leverage graph architecture in emotion recognition and related applications, however, their objective is not to exploit contagion between people but to extract complex relations between different modalities. Graph Neural Networks (GNN) are a popular architecture^25–27^ that have recently gained a lot of attention in healthcare and mental health applications^28–30^. In all these works^28–31^, graph representation is used to overcome the limitations of hand-crafted features with the objective of feature engineering. Our objective, on the other hand, is to exploit graph architecture to capture the role played by the users’ surroundings in their emotional state. Graphs created in previous related work are based on data from the *same* user. However, graphs created in this work are composed of *multiple* users.

In this work, we develop an emotion prediction model, Graph-based Emotion Recognition with Integrated Dynamic Social Network by integrating both temporal and spatial dynamics of physiological, behavioral, and social interaction information with graph convolutional neural networks and long short-term memory networks. Inspired by the concept of emotion contagion, we exploit user’s social networks based on call and sms logs and design graphs where users act as nodes and call and sms interactions between them are quantified as connectivity links. When information aggregation from other users in a participant’s network is conducted in an automated way, a limitation is imposed on the size of the input graph: it should stay fixed. However, in the real world, network size can change dynamically. Graph convolutional network (GCN) in a supervised node classification problem cannot handle this dynamic user distribution. The graphs in the dataset whose size is not equal to the model’s required input size cannot be fed to the model. If the graph size is larger than the required size, it can be converted to the required size by discarding some users but that results in a loss of information. To overcome this problem, we present GEDD: Graph Extraction for Dynamic Distribution. Inspired by the information aggregation mechanism in GCN, our method leverages graph properties like connectivity and components to transform the set of varying-size graphs into a set of graphs with fixed predetermined sizes. The proposed algorithm ensures that no users are discarded, and information is utilized to its full extent. Furthermore, it facilitates online learning with graphs where graph sizes are often changing. We develop an architecture that facilitates the integration of a user’s social dynamics in his/her emotion prediction such that,

- The sub-components of architecture are composed of GCN and LSTM layers that are differentiable and therefore can be easily trained using gradient descent methods.
- Extraction of graphs from existing phone data is automated.
- The architecture can adapt to the dynamic size of the user’s social network, which might change with time, allowing it to break extremely large networks into smaller networks in an efficient manner without any loss of information. With Internet-of-Things (IoT) networks and ubiquitous sensing, emotion recognition applications can be explored for macroscale networks and the proposed architecture can significantly facilitate these applications.

**Figure 1:**
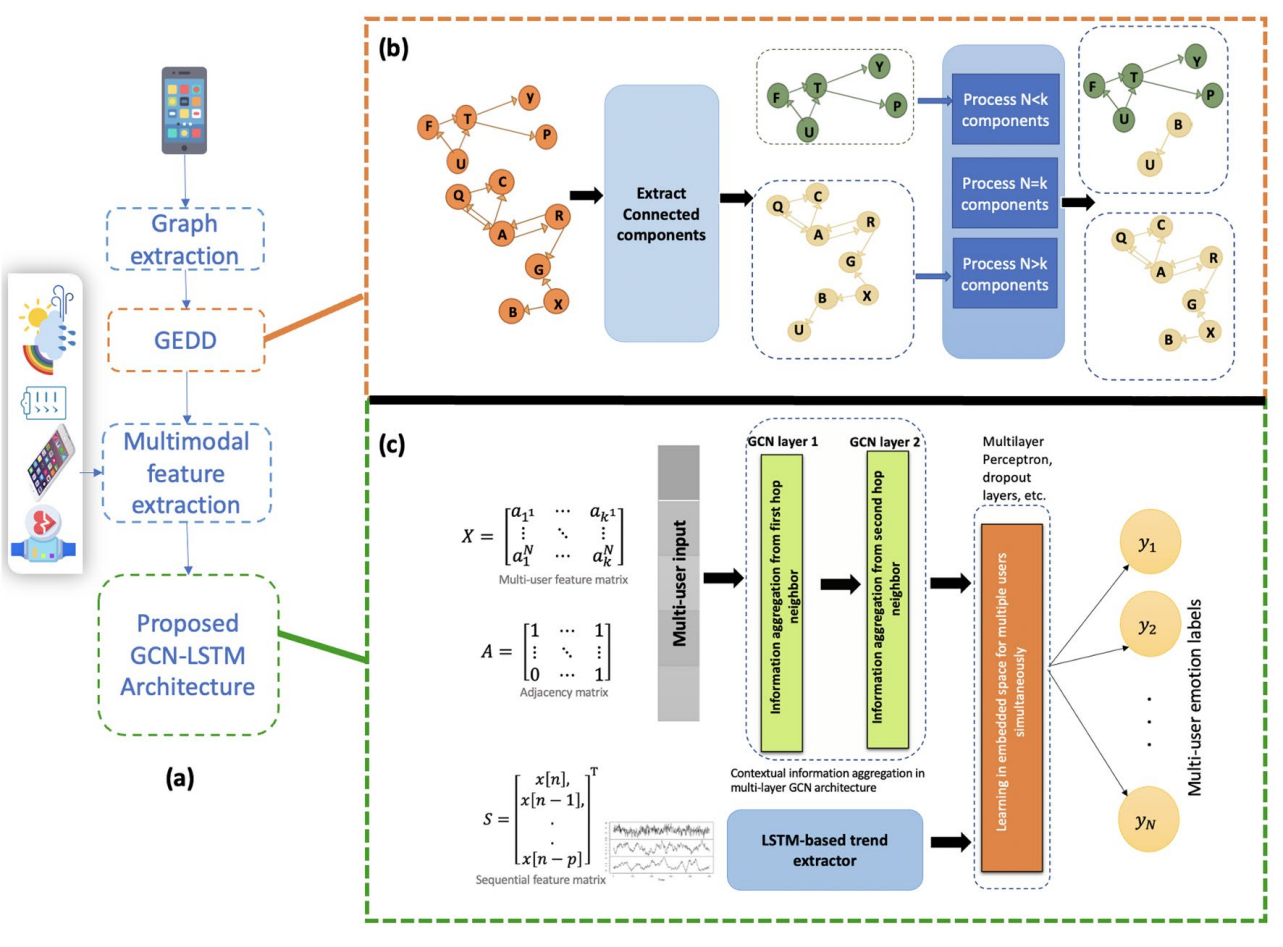
Graph-based Emotion Recognition with Integrated Dynamic Social Network architecture overview (**a**) Multi-user Graph-based learning flow diagram (**b**) Graph Extraction for Dynamic Distribution (GEDD) converts varying size graphs to the desired size with minimal loss of information (**c**) Graphical structure is integrated with temporal sensor data in the proposed Graph convolutional network-long-short term neural network (GCN-LSTM) architecture. The figure was drawn by the corresponding author in Microsoft PowerPoint, version 16.70 available at https://www.microsoft.com/en-us/microsoft-365/powerpoint.

The flow diagram for the proposed method is presented in Fig. 1a. To this end, we create multiple graphs from phone data and represent them by the adjacency matrix *A*. These extracted graphs are processed in the algorithm called Graph Extraction for Dynamic Distribution (GEDD) as shown in Fig. 1b. The algorithm is fed with the extracted graph *A* and model input size *w*. The algorithm breaks *A* into sub-components and processes them to provide multiple subgraphs each having *w* nodes (See details in “Methods”/“Graph extraction for dynamic distribution of users”). The adjacency matrices processed through GEDD are integrated with features in the multi-layer GCN module in Fig.1c. The spatial and temporal dynamics are integrated through concatenation and batch normalization layers and finally fed to the dense layer. We label the final model GCN-LSTM for ease of notation.

We test our models with the dataset collected from over 200 college students who were socially connected as participants in 30–90 day seven cohort studies. The data collected in the study include (i) mobile phone data (call and sms logs, GPS, and screen usage), (ii) physiology (skin conductance (SC), skin temperature (ST), and 3-axis acceleration (AC)), (iii) surveys (daily emotions, drugs & alcohol intake, sleep time, naps, exercise, academic and extracurricular activities) and (iv) weather (air pressure, humidity, wind speed, temperature, etc).

In an extensive experimental evaluation, the proposed model demonstrates improvement in emotion prediction accuracy resulting from the integration of user-to-user social interaction. The evaluation further explores the impact of network size that indicates the maximum neighbors taken into account by the model during prediction. The results indicate an initial improvement in accuracy with increasing network size until a plateau is reached after which diminishing returns are observed. This points toward the intricate dependency between graph topology, emotion, and prediction performance. To further gain insight into this conundrum, we conduct statistical analyses between the influence of users in the network, their emotional state, and the accuracy of the model. Our findings indicate a dependency of both, the emotional state and prediction error, on the eigenvalue centrality of the user.

## Results

### Quantifying mood and stress prediction improvement provided by network integration

We conduct an experimental performance evaluation to highlight the improvement in prediction provided by the aggregation of multiple users through a graph architecture. We hypothesize that the integration of social interactions through a graph structure improves the prediction performance quantified by the F1 score. To test this hypothesis, we compare our proposed GCN-LSTM model with equivalent models that are similar in all other aspects except the ability to incorporate graph structure in the same experimental setting. For comparison, we consider two other models that utilize the same multi-user input feature data but do not account for graph structure in the training/prediction:

- *LSTM only* In order to observe the improvement provided by graph integration, we also evaluate the model with LSTM layers only and no GCN. Like the proposed model, the LSTM layer is followed by multiple dense and dropout layers.
- *CONV-LSTM* In recent work^24^, the convolutional neural network was utilized to aggregate information from multiple modalities. For temporal dynamics, LSTM was used followed by dense and dropout layers.

We design the experiment to evaluate the performance and robustness of the proposed scheme. We account for sensitivity to initialization and generalization in our experiment design. After preprocessing (details in the “Methods” section), we train and test the models multiple times such that all three models are trained and tested on exactly the sample data samples within a trial. To depict the stability of the model, we use bootstrapping^32^ (more details in the “Methods” section) and report the average results along with the standard deviation across trials using micro-F1 score as the performance metric. The models input 314 features and predict the self-reported label (3 classes: low, mid, high) for stress and happy-sad mood the next evening (see details in the “Methods” section).

The performance results for empirical evaluation are reported in Table 1. The F1 score and root mean square error (RMSE) for both stress and happiness indicate that the proposed GCN-LSTM model provides higher prediction accuracy and lower RMSE compared to the other two baselines. Please also note that the proposed model has a much lower variance in both RMSE and F1 compared to the baselines which are much more sensitive to the train/test split, initialization, and model hyperparameters. It is interesting to note that during stress prediction, the CONV-LSTM baseline predicts values that were outliers, and that leads to extremely large RMSE. However, when the F1 score is computed, the continuous scores are converted to categorical labels mitigating the huge impact of a few outliers on the overall metric. Additionally, the analysis of variance (ANOVA)^33^ test is conducted to ensure that the difference between the performance of all three models is statistically significant. It can be observed that all p-values reported in the description of Table 1 are below 0.05 indicating that prediction accuracy for the three models is statistically different.

To further test our hypothesis about social networks boosting prediction performance, we utilize the Tukey HSD post-hoc test for three cases. The results from the post-hoc analysis indicate that the population mean F1 score of the proposed model is higher than that of CONV-LSTM for both stress (Tukey HSD, p-value = 0.0003) and happiness (Tukey HSD, p-value = 0.0054). The proposed model also performs better than LSTM-only for both stress (Tukey HSD, p-value = 0) and happiness (Tukey HSD, p-value = 0.000). Furthermore, CONV-LSTM performs better than LSTM for both stress (Tukey HSD, p-value = 0.0023 ) and happiness (Tukey HSD, p-value = 0.004).

**Table 1:** Performance comparison between different models.

Model	Stress		Happiness	
	F1 ± sd	RMSE ± sd	F1 ± sd	RMSE ± sd
GCN-LSTM	$0.69 \pm 0.02$	18.3 ± 0.66	$0.72 \pm 0.02$	$17.3 \pm 0.8$
CONV-LSTM	$0.62\pm 0.03$	95.0 ± 88.5	$0.65 \pm 0.05$	$20.6 \pm 2.5$
LSTM	$0.57 \pm 0.03$	43.3 ± 8.1	$0.57 \pm 0.04$	$56.7 \pm 24.1$

ANOVA test is conducted for statistical significance between different models. The test showed that all means reported in the table are statistically different for both stress (F1 p-value = $7.5\times 10^{-8}$, RMSE p-value =$2.0\times 10^{-2}$) and happiness (F1 p-value =$2.0\times 10^{-6}$, RMSE p-value =$1.6\times 10^{-5}$ ).

### Impact of graph size and sequence length on prediction performance

We further evaluate model performance for varying network sizes and sequence lengths. In the first experiment, the input graph size is varied, and the sequence length is fixed. The input graph size represents the amount of multi-user information that the model is exposed to for each sample. For a fair comparison between the models, the input size is kept the same across all models i.e if a graph of size 10 is constructed and provided to the proposed method, then the features and labels of the same 10 users are provided to benchmark schemes as well. The train and test sets are kept fixed across all models.

**Figure 2:**
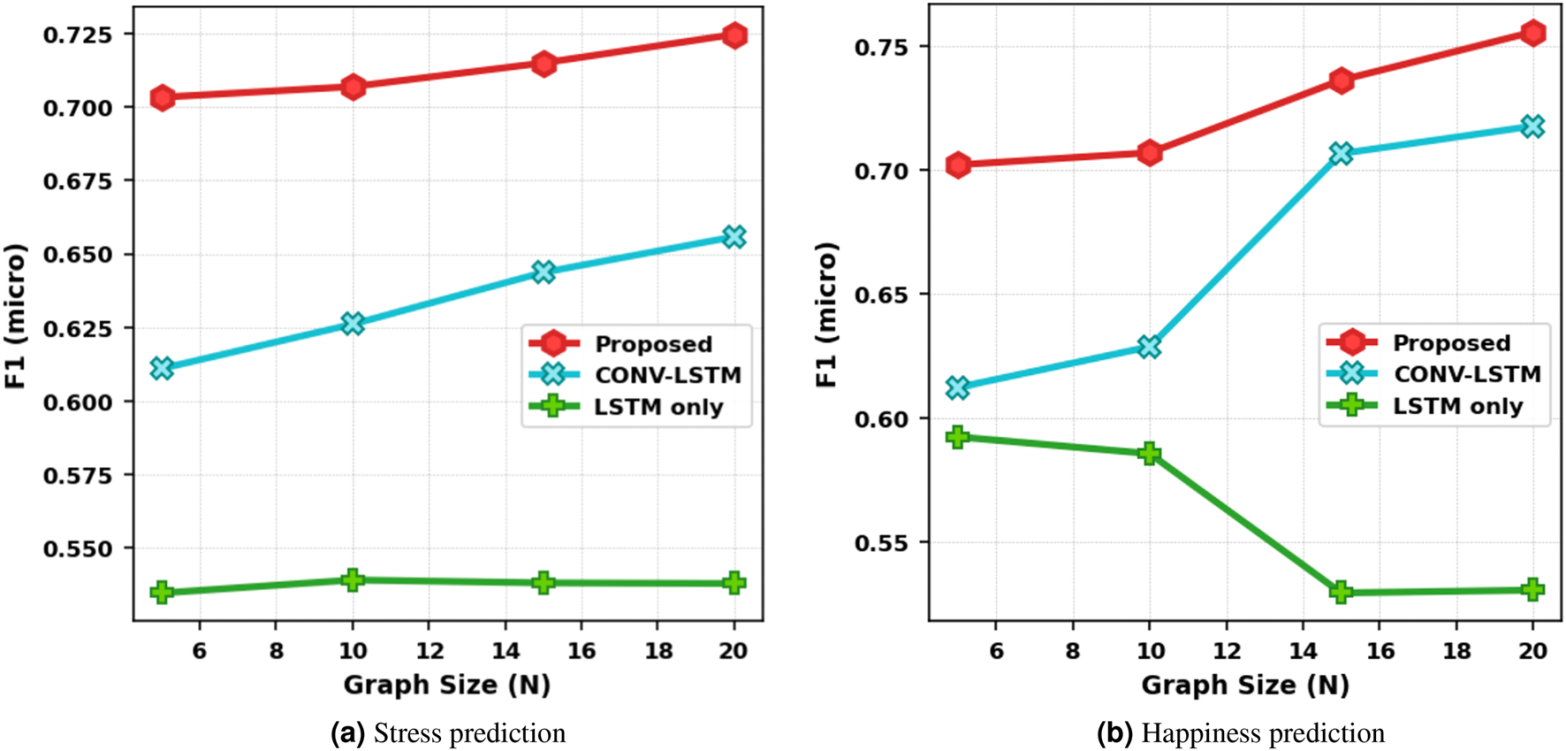
Impact of graph size on model prediction for fixed sequence length L = 5.

**Figure 3:**
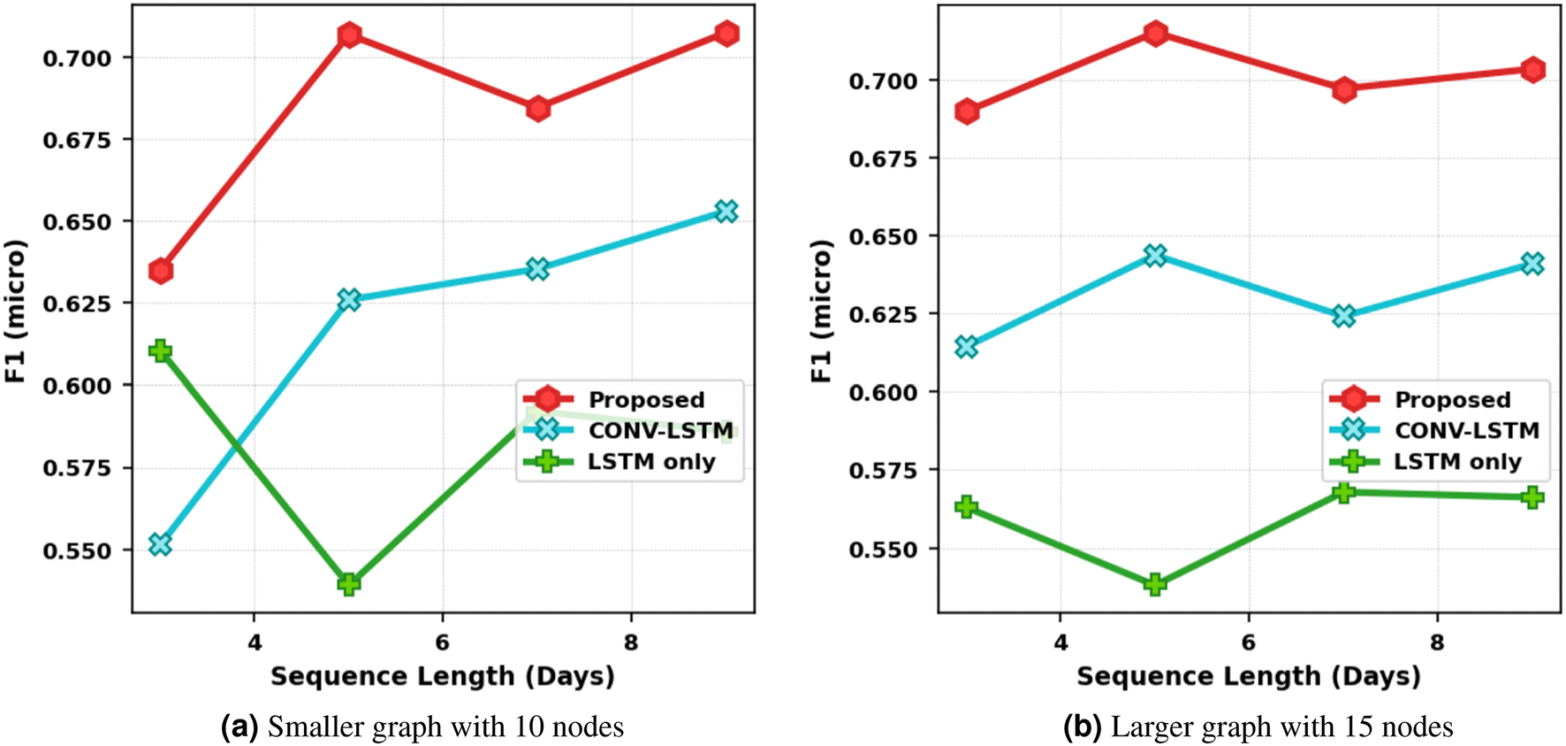
Impact of temporal memory on stress prediction.

The results for the impact of graph size on prediction accuracy are presented in Fig. 2. Figure 2a shows the F1 score for stress prediction and Figure 2b shows the performance for happiness prediction. Different lines represent the proposed method GCN-LSTM and benchmark models. First, we observe that for the proposed method and CONV-LSTM, an increase in graph size improves the performance. Even when the graph structure is not provided as in CONV-LSTM, a multi-user scenario is favorable for prediction compared to a single-user scenario. The slope of the proposed scheme is steeper in the beginning as we ascend towards larger graph sizes indicating a higher gain in performance. However, after N = 15, we see diminishing returns. This is because even though more nodes are added to the input graph, the edge density is becoming sparse, and therefore no additional information aggregation is happening. Comparing the three models in both plots, we observe that the proposed method outperforms the other two schemes by a significant margin highlighting the role played by the integration of social interactions in emotion prediction. Comparing the LSTM and CONV-LSTM, we observe that if aggregation between multiple users is not conducted in a systematic manner, it can be detrimental. The convolutional layer slides a filter across features to extract higher-level representations from those features^34^. When applied to a multi-user input, it treats the input as an image and extracts meaningful features that are fed into further layers. Thus, there is some level of systematic information aggregation happening compared to LSTM-Only which only extracts meaningful sequential information along the time axis. Therefore, LSTM-only performs worse than CONV-LSTM alone. Additional results for RMSE are presented in the supplementary information file in Fig. 3S.

We further investigate the impact of temporal memory on performance and present the results in Fig. 3. In this experiment, the graph size is kept fixed, and the time memory window is varied. Again, the number of input users is kept the same across all models and the same train/test data is provided for a fair comparison. Figure 3a presents the results for the small graph of size N = 10 and Fig. 3b for a larger graph N = 15 for stress prediction. Looking at individual plots, we observe that the GCN-LSTM method performs significantly better than LSTM alone and CONV-LSTM. When temporal memory is low with just 3 days of past feature data, the performance is also poor. However, when 5 days of past data is provided, we see a boost in the performance of all methods except LSTM-only. Further increasing the memory is detrimental as it introduces a false dependency on much earlier points in the past which actually do not play a part in the current emotional state. If we compare (a) and (b) in Fig. 3, L = 3 is an interesting point. For this short temporal memory, the CONV-LSTM performs worse than LSTM which is a deviation from the average trend and hypothesis analysis conducted in Table 1. However, when the input size is increased and multiple users are provided to the model at once, the model is able to *makeup* for the inability to capture temporal dynamics. Thus, we can conclude that the gap introduced by the unavailability of information provided by past data can be filled by utilizing the data from surrounding users.

The results for happiness prediction are presented in the supplementary information file in Fig. 2S. The performance improves with a larger graph size for all models. Similar to the trend observed in stress prediction, the performance improves when temporal memory is increased from 3 to 5 days and provides diminishing returns after that.

### Network characteristic analysis

To further gain insight into the emotion transfer dynamics between people, we investigate the impact of network behavior on prediction accuracy. The graph network is characterized by multiple factors derived from its nodes’ and edges’ attributes. The problem posed in this paper specifically focuses on contextual information aggregation from neighboring nodes and one key metric that can quantify this aggregation is node centrality/importance. After completing the learning pipeline (graph extraction, graph processing in GEDD, and training the model), we utilize multiple representations of node centrality including eigenvalue centrality, pagerank, degree centrality, and closeness centrality for the test dataset. The prediction accuracy of the model is determined by the average RMSE per user. To explore the relationship between node centrality (in graphs obtained from GEDD) and RMSE per user, the generalized estimating equations (GEE) method is utilized. GEE provides a mechanism to estimate the parameters of a linear model while accounting for correlation among different observations of a group. We preferred GEE to other statistical linear models like mixed linear-effects models because we are interested in the overall relationship between network topology and model performance that represents the *average* effect. Furthermore, GEE is robust to imprecise correlation structure. All centrality metrics are evaluated in the model together (details in “Methods”).

**Table 2:** GEE results with model prediction RMSE as dependent variable and node centrality metrics as independent variables.

	RMSE for stress prediction		RMSE for happiness prediction	
	Coefficient	p-value	Coefficient	p-value
Eigenvalue centrality	3.5	0.003	3.4	0.03
Small degree (D < 4)	− 1.5	0.02	− 1.6	0.02
Large degree (D > 4)	− 1.9	0.10	− 2.1	0.11
Closeness centrality	1	0.4	0.8	0.4
Pagerank centrality	− 0.008	0.6	− 0.01	0.6

The summarized results from the GEE analysis are shown in Table 2. The coefficients of the linear model between RMSE and node centrality metrics indicate the role played by that centrality in prediction and the p-value indicates the statistical significance of the learned coefficient. Please note that the results for closeness and pagerank centrality are not significant, so we can not use them for any inference. The degree centrality, which is the number of directly connected nodes, was categorized into two buckets: small degree and large degree with a threshold of 4 neighbors. For a small degree, we can observe that the coefficient is $-1.5$ indicating that the higher the degree, the lower the error because more information aggregation is happening. However, for large degree nodes, the results obtained are not statistically significant. This results from the tendency of the model to integrate information from irrelevant nodes during prediction. The same factors lead to a positive coefficient of 3.5 for eigenvalue centrality. Eigenvalue centrality assigns higher scores to nodes that are close to influential nodes. Thus, during the prediction of such nodes, the model aggregates information from a large number of nodes that connect to their neighbors. Since these are not direct neighbors, there is a high chance of incorporating information from unimportant nodes leading to higher RMSE.

GEE is also utilized to fit a model between true stress/happiness scores and the node centrality metrics. The results for stress and happiness are shown in Table 3 and supplementary information in Table 1S respectively. It can be observed that eigenvalue centrality plays a role in both emotions. Higher eigenvalue results in lower average mood scores and higher standard deviation in those scores. This standard deviation also explains why nodes that are connected to influential nodes have higher RMSE.

In summary, nodes with high eigenvalue centrality not only have lower happiness and calmness but also high variation in their emotion. Because these nodes have a large number of multi-hop connections, it is hard for the model to identify which neighbors are contagious and which are not leading to higher RMSE. The large variation in the emotional state is also a cause for higher RMSE for such nodes.

**Table 3:** GEE results with true stress score as dependent variable and graph centrality metrics as independent variables.

	Average stress score		Standard deviation in stress score	
	Coefficient	p-value	Coefficient	p-value
Eigenvalue centrality	− 7.1	0.002	3	0.03
Small degree (D < 4)	0.96	0.6	− 0.7	0.23
Large degree (D > 4)	− 1.8	0.4	− 1.3	0.31
Closeness centrality	0.5	0.9	− 0.6	0.8
Pagerank centrality	0.01	0.5	− 0.001	0.5

## Discussion

The deep learning architecture discussed in this work focuses on the prediction of multiple people’s emotions at the same time. Multi-user learning requires an informed mechanism to aggregate information from multiple users. It is intricate because there is a correlation between features, correlation within users, and correlation between features and users. Convolution layers in CONV-LSTM explore this correlation in a more systematic way than LSTM and therefore performs better. This also explains why sometimes LSTM has overshooting RMSE in prediction results presented in the Supplementary Section and thus a much higher standard deviation in error. The proposed model performs the best because it utilizes additional information about within-user dependency. The integration of this dependency resulting in better prediction accuracy substantiates the impact one person’s emotion has on the other.

The way graphs are constructed to incorporate emotion contagion is also critical. Even though the performance evaluation indicated an overall improvement in prediction with the integration of social graphs, the network characteristic analysis revealed that higher eigenvalue centrality and a very large degree (greater than 4) negatively impact the performance. The phone data can indicate interaction with a large number of people however not all of them play a critical role in that person’s emotions. Similarly, when people are connected to influential people, the machine learning model would integrate information from the neighbors of those influential people many of whom might not have a significant impact. To overcome this limitation in future works, hierarchical graph structures can be utilized to distinguish between contagious and irrelevant nodes. Furthermore, these findings suggest that more sophisticated graphs in an online learning framework like reinforcement learning should be explored in future research. Feedback, indicating which users are helpful for prediction during learning, would not only refine the graph and improve prediction accuracy but also yield deep insights into social contagion dynamics. With the availability of a huge amount of online data, the infrastructure proposed in this work, for constructing graphs and predicting emotions, is particularly useful for examining large-scale networks and therefore would be a resource for future research in macroscale social contagion.

The dataset used in this paper and other open-source emotion-related datasets, with wearable and mobile phone data, are not designed to capture the complete social network of the participants. There is a small likelihood that all friends and family of a participant are also participating in the same study. This is a major limitation since it results in sparse graphs and a significant amount of the impact made by a user’s surroundings on his emotional state is not captured. The evaluation results presented in this paper show that graph-based architecture always performs better than the architecture that does not account for it. However, there is still a large margin for improvement that graph-based architecture can provide if more dense graph networks are available.

Graphs provide a simple yet powerful tool to represent multi-user information. This work is limited to constructing graphs from call and SMS data only, because of data availability. Other social activity features such as in-app calls and chats were not collected. Location was collected which was utilized to create graphs and connect people who were in each other’s vicinity. However, the frequent indoor mobility of the participants made precise location tracking difficult. The resulting graphs were also extremely sparse and did not provide meaningful connections for multi-user information aggregation. Thus, graphs obtained from proximity were not utilized further. This study was not designed to study the effect of face-to-face social networks on emotion or other outcomes. In future works, along with social networks, graphs constructed from similar habits, physical proximity, mobility patterns, lifestyle, etc. can be integrated into the proposed architecture. The proposed model can handle both static and dynamic graphs.

Another limitation of this dataset is the low temporal resolution of social interaction. Since limited call and SMS logs are available, meaningful graphs can only be extracted when data spanning a time interval of a few days is considered. However, if more data is available for a larger number of participants, graphs can be extracted at hourly resolution and the prediction problem can also be solved at a higher temporal resolution for moment-to-moment emotion network contagion. The dataset was also limited to a few months of data for each participant which makes it difficult to quantify the difference between long-term^21^ and short-term contagion.

When establishing a graph network between users from call and SMS logs, we noticed that participants were connected across cohorts as well. When taken into account, that provided us with a global graph network. However, since wearable and mobile phone data for those out-of-cohort connections were not available for the same time interval, we were not able to utilize them. In future works, algorithms for network sampling, estimation, and inference can be deployed to overcome this limitation and both global and local graph structures can be combined to improve the prediction performance.

One major challenge in emotion recognition problems is the collection of ground truth emotional state. There is no sensor to directly measure happiness or sadness. Therefore, we must rely on an individual’s judgment of their emotion which leads us to the second challenge associated with this problem: it is a user-centric study. Since the ground truth is collected through ecological momentary assessments (EMA), it is a tedious and expensive process. As graphs provide a global view of multiple connected users, they can be leveraged to identify *important* users whose data would be most beneficial for the overall prediction accuracy of the whole network. Once important users are identified, EMA data is collected from only them instead of the entire network.

The proposed work has significant utilization in real-world applications ranging from recommendation and regulation systems to web customization. A large amount of research work has focused on optimizing lifestyle by regulating activity, eating habits, and sleep schedule^35,36^. However, little attention has been paid to improving mental health by regulating social interactions and this work can play a pivotal role in such applications. This social interaction management can be taken a step further by developing apps that can customize the social media experience. Furthermore, the architecture proposed in this work can prove particularly useful in investigating emotion dynamics of people who spend a large amount of time as part of special environments such as healthcare workers, caregivers, rehabilitation counselors, etc.

## Methods

### Data collection and processing

We utilize a multimodal dataset collected in 2013–2017 from college students (age: 17–28, 146 male and 80 female) who were socially connected as participants. The study was conducted over multiple different time periods during each academic term for 4 years. Different students were recruited for 30–90 day studies in each academic term (N = 20–113 each in seven cohorts). Four different types of data were collected in the study. After pre-processing of data, there are 314 features. Please see details about features and labels in the Supplementary document.

- *Mobile phone data* An app was installed on the participant’s phone that recorded the call logs, SMS logs, GPS, and screen usage along with timestamps. For phone features, statistics such as the mean, median, and frequency of these phone usage data were calculated for each time period (0–24 h, 0–3 h, 3–10 h, 10–17 h, 17–24 h). Also, mobility features such as total daily distance, time spent on campus, and time with indoor/outdoor indications were calculated.
- *Physiological data* From wearables, electrodermal activity (EDA) measured as skin conductance (SC), skin temperature (ST), and 3-axis acceleration (AC) were collected at 8 Hz. For each time period (0–24 h, 0–3 h, 3–10 h, 10–17 h, 17–24 h), we calculated features about SC peaks and levels, ST, AC, and combinations of these physiological data streams.
- *Surveys* Online surveys were filled by participants each morning and evening and contained information about drugs & alcohol intake, sleep time, naps, exercise, and academic and extracurricular activities. All users filled out a survey indicating their calmness (stress) and happy-sad mood on a non-numeric scale (scored 0-100) every day. We use these scores as ground truth in our problem. The exact question asked is as follows:
- *For each of the following, indicate how you feel right now by clicking on each line and adjusting the sliders.*
  - *Sad - Happy*
  - *Stressed Out - Calm Relaxed*
- *Weather* Data about weather conditions was extracted from Sky web API^37^ which was processed to extract air pressure, humidity, wind speed, temperature, information about sunlight and moon phase, and daily weather deviation from the rolling average.

The empirical distribution of both happy-sad mood and stress follows a Gaussian function. The mean and standard deviation for mood score across all samples is 61.8 and 23.8. The mean and standard deviation for stress score across all samples is 54.0 and 26.0. A detailed description of the input features and their descriptive statistics are provided in the Supplementary document Sections 2.3 and 2.4.

### Graph-based stress and mood prediction models

The objective of this work is to predict the emotional state (stress and happy-sad mood) of a user based on multimodal data collected from the wearable, mobile phone, and user-reported survey data. We focus on well-being prediction in terms of stress and mood. The well-being metric for each day *n* is represented as $y[n] \in [0,100]$. Since the labels are not available at a frequency higher than 1 per day, we project the daily multimodal data to a compact representation such that for each feature we have one value per day by taking the mean and variance for different intervals of the day. For ease of understanding, we represent this compact feature data for the day *n* in form of a vector *x*[*n*].

For each *n*, the objective is to utilize the past *l* days of information to predict *y*[*n*],

1 $$\begin{aligned} {\hat{y}}[n] = \underset{\theta }{\textrm{argmin}} \;\; \Vert y[n]- f( \varvec{X_l^{n-1}},\theta )\Vert _2^2 \end{aligned}$$

where $\theta$ represents the model parameters and *l* represents the time steps (memory) that model takes into account for prediction,

2 $$\begin{aligned} \varvec{X_l^{n-1}}=[x[n-1], x[n-2], \ldots ,x[n-l-1] ] \end{aligned}$$

Taking the mean and variance of different intervals of the day and representing each interval as a separate feature results in a huge loss of information. To reduce this loss, we deduce knowledge about how well users are connected from this data and utilize it to improve our model. We leverage graph networks to indicate the connectivity of participants (see more details about graph construction in the next subsection). We develop a weighted graph network ${\mathscr {G}}$ between a set of participants/users ${\mathscr {V}}$ whose connectivity or closeness is represented by a set of edges ${\mathscr {E}}$,

$$\begin{aligned} \mathscr {G}=(\mathscr {V},\mathscr {E}) \end{aligned}$$

We can represent this graph as adjacency matrix ${\varvec{A}}$ where the value at *i*th row and *j*th column is represented by $A_{ij}$,

3 $$\begin{aligned} A_{ij} = {\left\{ \begin{array}{ll} w_{ij} &{} \text {an edge} \,{\mathscr {E}}_{ij}\,\text {exists \, from} {\mathscr {V}}_i \, \text {to}\, {\mathscr {V}}_j \\ 0 &{} \text {otherwise} \end{array}\right. } \end{aligned}$$

And $w_{ij}$ represents the weight of edge. The objective of the model is to predict ${\hat{y}}[n]$ with minimum error,

4 $$\begin{aligned} {\hat{y}}[n] = \underset{\theta }{\textrm{argmin}} \;\; \Vert ||y[n]- f( \varvec{X_l^{n-1}},{\varvec{A}},\theta )\Vert _2^2 \end{aligned}$$

### Graph extraction

The social interactions between participants are captured through a graph network. A graph is composed of two main components: nodes and edges^38^. Node is a vertex that is connected to other vertices through lines called edges. In some problem settings such as social media networks, it is straightforward to establish a link e.g., when two users are friends, they are connected. However, if social media information is not collected and participants of a study do not provide information about whether they are friends with each other, creating edges is not straightforward. Even if users indicate friendship, as in the latter case, we need to define a graph that is most helpful in achieving the prediction objective. Please note that one-time survey to identify whether users are friends with each other is not sufficient because they might be friends, but they do not interact very often because of different class schedules or circumstances. Instead, we utilize the call and text message exchanges to establish weighted links between users as shown in Fig. 1a. We create two graphs: call graph ${\mathscr {G}}_c$ represented by adjacency matrix $\varvec{A^c}$, and SMS graph ${\mathscr {G}}_s$ represented by $\varvec{A^s}$. We design them based on phone data collected over an interval [0, *T*]. Representing an incoming call of duration *d* from user ${\mathscr {V}}_i$ to ${\mathscr {V}}_j$ at time *t* by $C_{ij}[t]$,

5 $$\begin{aligned} C_{ij}[t]= & {} {\left\{ \begin{array}{ll} d &{} {\mathscr {V}}_i \,\text {calls} \,{\mathscr {V}}_j\\ 0 &{} \text {otherwise} \end{array}\right. } \nonumber \\ A^c_{ij}= & {} \sum _{t=0}^{T}C_{ij}[t] \end{aligned}$$

For text messages, we consider two types of incoming messages: normal SMS with a text message body (Class 1 message), and flash SMS with no message body (Class 0 message). Denoting an SMS from user ${\mathscr {V}}_i$ to ${\mathscr {V}}_j$ at time *t* by $S_{ij}[t]$,

$$\begin{aligned} S_{ij}[t] = {\left\{ \begin{array}{ll} w_1 &{} {\mathscr {V}}_{i} \, \text {sends} \,{\mathscr {V}}_{j} \, \text {a Class 1 message}\\ w_2 &{} {\mathscr {V}}_{i} \,\text {sends} \,{\mathscr {V}}_{j} \,\text {a Class 0 message}\\ 0 &{} \text {otherwise} \end{array}\right. } \end{aligned}$$

Prioritizing Class 1 messages because of their stronger interaction $w_1 > w_2$, we construct SMS graph,

6 $$\begin{aligned} A^s_{ij} = \sum _{t=0}^{T}S_{ij}[t] \end{aligned}$$

For each node in the adjacency matrix, there is associated feature data *X* and label data *y*. The time interval *T* is equal to each cohort’s study interval which is approximately equal to a few months.

### Contextual aggregation from multiple users

When considering a multi-user scenario, there are two further sources of information that need to be exploited to make predictions about emotional state. The first source is the individual’s feature vector *X*, computed from the mobile phone, wearable, and survey data. The second is the relationship between multiple users i.e. the structural properties of the graph. When considering feature data for each user independently, neural networks can learn the underlying model and provide predictions. Further improvement can be made by exploiting local structure by deploying convolutional neural networks that use kernels/filters to extract complex features from a grid-like structure^39^. However, they cannot be utilized in this problem because they cannot operate on a graph-like structure. The flattened adjacency matrix cannot be utilized as input to these models because the neural network is not permutation invariant i.e it depends on the ordering of nodes in the adjacency matrix. This problem is addressed by GCN proposed in^40^.

Inspired by spectral convolutions on graphs, GCN provides a layer-wise linear propagation rule that allows a neural network to learn from graphs. Spectral graph convolution is the convolution of any signal *x* with a filter *g*, where the filter *g* is derived from the graph^41^. In order to compute spectral convolution, we need the laplacian and degree matrix of a graph. The degree matrix *D* is a diagonal matrix containing degrees of the nodes on the diagonal where degree of a node is sum of incident edges.

For more details on GCN layer, please refer to supplementary information Section 3 and related work^40,42^. The forward propagation at layer *f*th of multi-layer graph convolutional neural network is represented by $H^f$ ,

7 $$\begin{aligned} H^f = \sigma ( {\tilde{D}}^{-1/2} {\tilde{A}} {\tilde{D}}^{-1/2} H^{(f-1)}\Theta ^f ) \end{aligned}$$

where $H^f$ represents *f*th layer of GCN, ${\tilde{A}}$ represents the graph adjacency matrix, $\Theta ^f$ are the weights of *f*th layer and $\sigma$ is the activation. The input to the first layer is the node feature matrix *X* defined in Eq. (2). This equation is very similar to that of a dense layer of a conventional neural network except that the degree and adjacency matrix representing the graph aggregate the inputs from previous layers based on user connectivity.

To predict well-being labels using this model, labels for each user/node are used to compute the cross-entropy loss, and the model parameters are learned through forward and backpropagation. The training process and parameter tuning are similar to the conventional neural networks. The only difference is that in addition to the feature matrix *X*, graph adjacency matrix ${\tilde{A}}$ is also computed through call and SMS interaction data in our project and used as input to the model.

The graphs extracted for different cohorts vary in size in the range 20–50 nodes. The minimum and maximum number of directly connected neighbors of a given participant are 0 and 12 respectively. The average number of direct neighbors across all cohorts is 1.2 with a standard deviation of 2.2.

**Figure 4:**
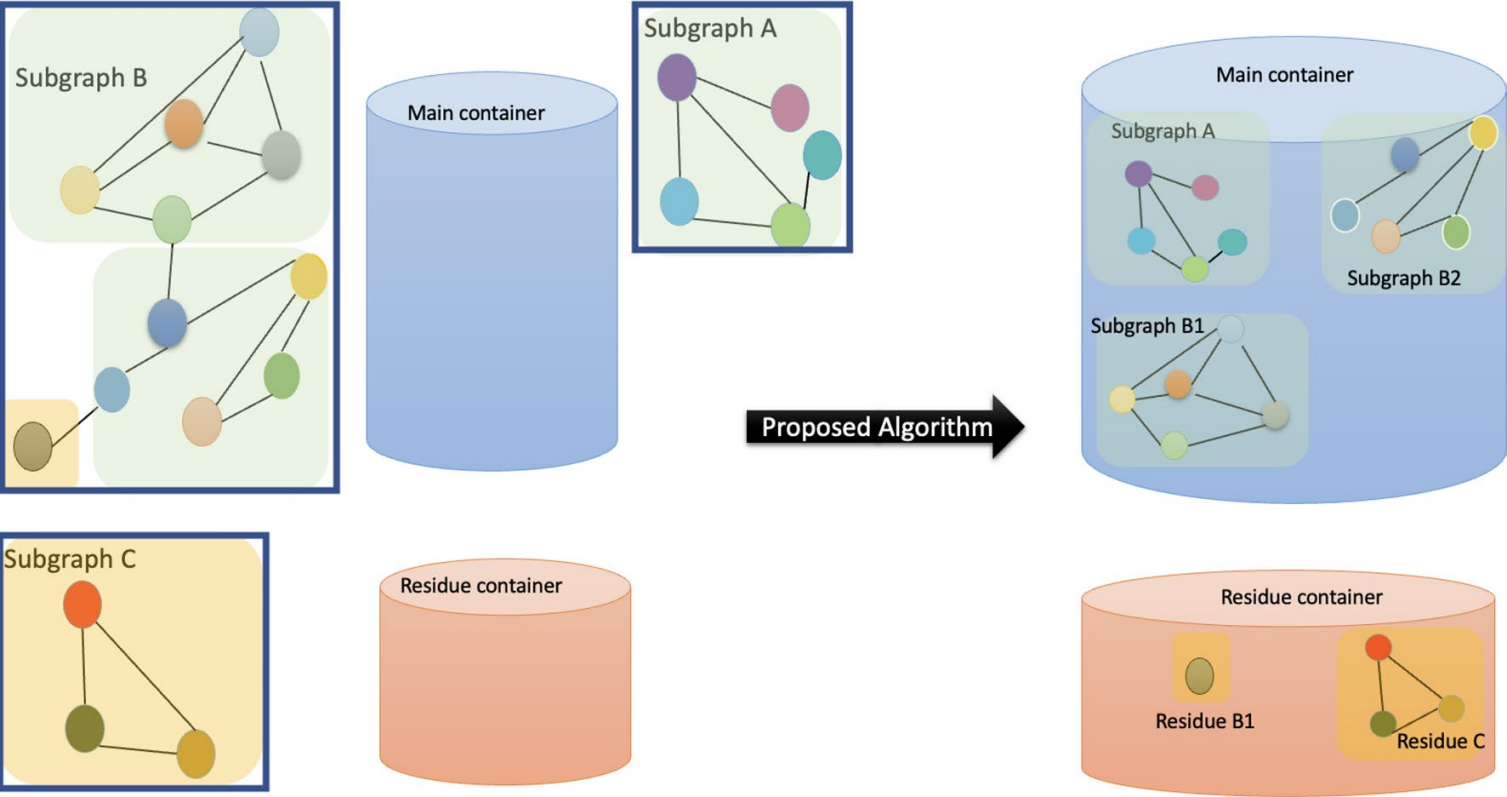
Framework to extract subgraphs of size *w* for dynamic user data. The proposed algorithm GEDD first converts large components into smaller subgraphs of size *w* with a residue. The optimal and adjusted graphs are added to the main container and the residue subgraphs of size $<w$ are to the residual container. In the end, the residual graphs are processed to obtain the required size *w* graphs. The figure was drawn by the corresponding author in Microsoft PowerPoint, version 16.70 available at https://www.microsoft.com/en-us/microsoft-365/powerpoint.

### Learning temporal dynamics

When utilizing multi-modal data for well-being prediction, it is important to realize that most well-being indicators are not impulsive and independent entities. Several factors lead to a certain state. It is very likely, that evening health conditions could not be explained by the data collected in the morning but from the temporal dynamics of the same features for the past few days. To integrate these dynamics into the model, an element of ’memory’ is required that can utilize contextual information to predict future emotional states.

For the emotion prediction problem, we extract the sequential information in features to predict the well-being label *y*. For a given user, let $x[n]\in {\mathbb {R}}^N$ and *y*[*n*] represent the feature vector and well-being score for day *n* respectively. If $p\in {\mathbb {Z}}^+$ is the length of the sequence, then we create an $N\times p$ sequence matrix,

8 $$\begin{aligned} S_n^p =\big [x[n-p]],x[n-p+1], \ldots , x[n]\big ] \end{aligned}$$

This sequence matrix serves as an input feature matrix for which we predict the future well-being label $y[n+l]$. Here $l\in {\mathbb {Z}}^+$ represents how far in the future we want to make the prediction. The tuples $(S_n^p,y[n+l])$ are used to train an LSTM network in a supervised fashion with the cross-entropy loss function. The model weights are learned through conventional forward and backward pass over the model with gradient descent.

### Graph extraction for dynamic distribution of users

The data were collected from different sets of socially connected participants in each cohort. Each cohort has a different number of participants who enrolled in the study on slightly different study start and end dates. The variation in the number of participants per cohort is challenging because the proposed GCN-LSTM model requires both features and graph network as input. Both these inputs *fix* the size of the input layer. When the number of users changes, the size of the graph changes from the model’s predetermined input size. Conventional methods used in image classification such as padding, or truncation are not feasible because graphs are different from images and have a more non-uniform structure. Truncation of such a structure would result in a huge loss of information. When a node is discarded from the graph, not only the graph structure information is lost but also the user’s feature matrix comprising a large amount of physiological, mobile, and survey data.

To overcome this problem of varying user sizes, we propose an algorithm called Graph Extraction for Dynamic Distribution (GEDD). It is a connected component-based method that converts large dynamic graphs into a set of small graphs of size equal to the model’s input size *w*. Our method is inspired by the working principle of the graph convolution network presented in Eq. (7). GCN exploits graph structure by combining a node’s feature vector with its neighbor’s information. In the first layer, information from one-hop neighbors is aggregated. In the second layer, information from 2-hop neighbors is integrated. As we go deep down the network, the knowledge from farther away neighbors is aggregated. Therefore, for label prediction of a given node $w_i$, a node $w_j, j\ne i$ would only contribute if it is connected directly or through a multi-hop connection to $w_i$, and nodes that are not connected are irrelevant for $w_i$’s prediction.

We exploit this concept for extracting graphs of size *w* by utilizing connected components. A connected component of a graph is a subgraph in which each node is connected to another through a path^38^. For a graph with *N* nodes $\mathscr {G}_\mathscr {N}=(\mathscr {V},\mathscr {E})$, there are *p* connected components with $1\le p\le N$. When $p=1$, all the nodes in $\mathscr {G}_\mathscr {N}$ are connected, and when $p=N$, all nodes are disconnected and have 0 degree. The breakdown of graph in connected components will result in subgraphs of varying sizes. Let $C_i$ represent the *i*th connected component,

$$\begin{aligned} C_i = \{{\mathscr {V}}^j,{\mathscr {E}}^j\} \;\; i={1,2, \ldots p}\;j={1,2, \ldots N} \end{aligned}$$

and $\Vert C_i\Vert =q_i, 1\le q_i \le N$ represent the size of the component. First, the components are divided into two containers, Main container ${\mathbb {M}}$ and residue container ${\mathbb {R}}$, based on their size as shown in Fig. 4. The former will contain subgraphs of size *w* and the residual will contain graphs of size $r< w$. This leads to three scenarios,

- when $q_i=w$, add $C_i$ to ${\mathbb {M}}$
- when $q_i<w$, add $C_i$ to ${\mathbb {R}}$
- when $q_i>w$, break $C_i$ into $j=\lceil \frac{q_i}{w} \rceil$ subgraphs $C_i^b$ where $b={1,2, \ldots j}$. The large component is broken such that,
  $$\begin{aligned} C_i^b = {\left\{ \begin{array}{ll} w &{} b={1,2, \ldots ,j-1}\\ q_i \bmod w &{} b=j \end{array}\right. } \end{aligned}$$
  The subgraphs that satisfy first condition in above equation $\{C_i^1,C_i^2, \ldots ,C_i^{j-1}\}$ are added to ${\mathbb {M}}$ and $C_i^j$ to ${\mathbb {R}}$.

Once the components are divided between two containers, the main container is ready to be fed to the model. For the residue container with all subgraphs smaller than *w*, we concatenate multiple subgraphs to create size *w* subgraphs. There is still some residue left at the end when the *total* number of nodes in ${\mathbb {R}}$ are less than *w*. For this last set, we use repetition of nodes to create a final size *w* subgraph.

### Machine learning pipeline

We design the experiment to evaluate the performance and robustness of the proposed scheme and account for sensitivity to initialization and generalization in this process.

#### Preprocessing

The pre-processing of data involves two main steps, dealing with missing data and standardization. First, features with more than $\lambda /2$ missing values are removed, where $\lambda$ is the total number of samples. For the remaining features, the filling data is filled in two main steps. In the first step, the missing entries for a given user are filled using its own data through k-nearest neighbor imputation. This method identifies k neighbors for a datapoint and replaced the missing value with the mean value of those neighbors. In the second step, we consider all users together and repeat the k-nearest neighbor imputation for the entire dataset. Finally, we detect outliers using z-score statistics and remove them. Z-score is computed by subtracting from the datapoint it’s mean and dividing by the standard deviation.

As mentioned in the dataset description section above, the feature matrix is composed of multiple modalities that come from very different distributions. In order to account for the difference between features’ scale and spread, we standardize the data i.e we transform the distribution of data such that it has a 0 mean and unit standard deviation. To achieve this transformation, first, the mean of data is subtracted from it and then this zero-mean data is divided by its standard deviation.

#### Performance metrics

The model predicts the label for stress or happy-sad mood. The score for mood is categorized into three bins with class labels 0, 1 and 2. Class 0 indicates high stress/low happiness ($\le 33$), class 1 indicates moderate stress/happiness, and class 2 indicates low stress/high happiness ($\ge 66$). The participants fill out a survey every day about how happy and stressed they are on a scale of 1–100 which is utilized as ground truth for the following experiments.

Since this is a multi-class problem, we utilize the F1 score as the performance metric. Moreover, since the problem is multi-class and the classes are imbalanced, we weigh all classes accordingly and therefore use a micro-average F1 score. The proportion of class labels 0, 1 and 2 for happiness is $12\%$, $46\%$, and $42\%$ respectively. The proportion of class labels 0,1 and 2 for stress is $23\%$, $46\%$, and $31\%$ respectively. To compute the F1, first we calculate micro-average precision *P* and recall *R*,

9 $$\begin{aligned} P = \frac{\sum _{p=1}^{3} TP_p }{\sum _{p=1}^{3} TP_p + FP_p } \end{aligned}$$

10 $$\begin{aligned} R = \frac{\sum _{p=1}^{3} TP_p }{\sum _{p=1}^{3} TP_p + FN_p } \end{aligned}$$

where $TP_p$, $FP_p$, and $FN_p$ represent true positives, false positives, and false negatives for class *p* respectively and *p* denotes the class ID. Finally, the micro-average F1 score is computed as follows,

11 $$\begin{aligned} F1 = 2* \frac{P*R}{P+R} \end{aligned}$$

#### Bootstrapping

In order to remove the impact of the sensitivity of the model to parameter initialization, we repeat the training and testing procedure ten times and report average results along with the standard deviation across trials. Furthermore, the size of the dataset is of the order of a few thousand which is small in comparison to feature space and model complexity. Therefore test-train split can impact the performance. To account for this, we split the data into test and train sets randomly and repeat the procedure ten times.

#### Model training

For learning the model parameters, $50\%$ of data is used for training, $10\%$ for validation, and the remaining for testing. The models are trained with input data and sms graphs, and for training, we utilize ADAM optimizer with the same learning rate for all three models. During the training procedure, validation loss is monitored, and the model comes to an early stop if validation loss is not changing by more than $\Delta =10^{-5}$ for more than 50 iterations. This helps save computational time and cost.

### Network characteristic analysis

#### Network characteristics

Graph network is not only indicative of user clusters but also complex interconnectivity. The notion of connectivity has many aspects which are captured by different types of centrality metrics defined in literature^38^. We hypothesize that the more central a user is in the network, the more information aggregation would happen in the prediction model and that would impact the model prediction performance. The centrality metrics used to quantify the influence of a person in the network are listed below,

- *Degree centrality* is a direct representation of how many directly connected neighbors a node has. If the graph is represented by $N\times N$ adjacency matrix *A*, then the degree centrality $C_d$ of a node *v* is calculated as,
  12 $$\begin{aligned} C_d(v) = \sum _{u=1}^{N}{\mathscr {I}}(A_{uv}) \end{aligned}$$
  where,
  13 $$\begin{aligned} {\mathscr {I}}(x) = {\left\{ \begin{array}{ll} 1 &{} \text {if } x > 0\\ 0 &{} \text {x = 0} \end{array}\right. } \end{aligned}$$
  Degree centrality assigns higher importance to nodes that have a large number of neighbors. However, it does not account for the cascade effect resulting from the fact that a node can also be important if it is connected to influential nodes.
- *Closeness centrality* represents the importance as to how close a node is to other nodes in terms of geodesic distance. To compute the closeness centrality $C_c$ of a node *v*, the shortest distance to all other nodes is computed,
  14 $$\begin{aligned} C_c(v) = \sum _{u\in {\mathscr {V}}}\frac{N}{ d(u,v)} \end{aligned}$$
  where *d*(*u*, *v*) is the shortest path from node *v* to *u*. This is computed using Dijkstra’s algorithm^43^.
- *Eigenvalue centrality* quantifies the influence of a node in a network by measuring the node’s closeness to influential parts of a network. It combines the degree of a node with the degree of its neighbors. For a graph ${\mathscr {G}}$ with adjacency matrix *A*, the eigenvalue centrality $C_e$ of a node *v* is calculated by^44^,
  15 $$\begin{aligned} C_e (v)= \alpha \sum _{u,v\in {\mathscr {E}}}C_e(u) \end{aligned}$$
  where $C_e$ and $1/\alpha$ are the eigenvector and corresponding eigenvalue of *A* respectively,
  16 $$\begin{aligned} AC_e = \alpha ^{-1}C_e \end{aligned}$$
  Please note that solving the eigenvalue problem for large graphs is expensive. In this scenario, the power iterations method is used to compute the eigenvalue and the corresponding eigenvector for a graph with *N* nodes and ${\mathscr {O}}(N_v^2)$ complexity,
  17 $$\begin{aligned} C_e(z+1) = \frac{AC_e(z)}{\Vert | AC_e(z)\Vert |} \end{aligned}$$
  where *z* represents the iteration index.
- *Pagerank centrality* assigns high importance to nodes that are connected to important nodes, or if they are linked by a lot of other nodes who themselves have small outgoing connections. Thus, it incorporates both, the importance of neighboring nodes and the number of incoming edges. It was proposed by^45^ to retrieve relevant pages from the web in response to a query. To calculate pagerank centrality $C_p$ for adjacency matrix *A* , first, the in-degree $d^{in}$ and out-degree $d^{out}$ is calculated for node *v*,
  $$\begin{aligned} d^{in}(v) = \sum _{u=1}^{N}A_{uv}, \;\;\;\;\;\;\;\;\;\;\; d^{out}(v) = \sum _{u=1}^{N}A_{vu} \end{aligned}$$
  18 $$\begin{aligned} C_p(v ) = \gamma \sum _{u=1}^{N}\frac{A_{uv}}{d_u^{out}}C_p(u) +\frac{1-\gamma }{N} \end{aligned}$$
  where *N* is the number of nodes of the graph and $\gamma$ is a constant damping factor. For nodes, with no outgoing links, the algorithm would get stuck and therefore, such nodes are known as sinking nodes. To avoid this problem, a damping factor is introduced that prevents the algorithm from terminating when ending in such sinking nodes.

#### Outcome metrics for statistical analysis

The overall evaluation metric for the model is RMSE. However, since the test samples are chosen randomly and the model is trained and evaluated multiple times, multiple predictions for different users with different graph characteristics are obtained. Furthermore, there are participants whose connectivity dynamics changed over time and so do the resulting centrality scores. While defining the outcome metric, it is important to ensure that the model distinguishes between the performances of the model for a participant in different graph topologies i.e. a user can have different prediction accuracy when its centrality changes. To achieve this, we define RMSE per user and compute multiple RMSE scores for the same user for different graph topologies. At the end of the multi-trial evaluation, we filter out the predictions for user *i* when it was in a topology *j* and create the column vector $y^p_{i,j}$ with true labels $y^t_{i,j}$ and compute the RMSE $R_i^j$,

19 $$\begin{aligned} R_i^j = \sqrt{\frac{\sum _{d=1}^{D}(y^p_{i,j}[d]- y^t_{i,j}[d] )^2}{D}} \end{aligned}$$

where *y*[*d*] denotes the $d^{th}$ entry of the vector *y* and *D* is the length of vector $y^p_{i,j}$.

#### Statistical model

After defining the independent variable representative of graph characteristics and dependent evaluation criteria, we investigate the relationship between them. We fit a linear marginal model between the two variables in a clustered data analysis setting while accounting for within-cluster and between-cluster variations.

Fitting the same model to all the participants and treating the whole population as one cluster would result in over-simplification of the underlying complex model. While the emotional state of a person is affected by his surroundings and social interactions, the magnitude, and type of this effect varies from person to person. We capture this individual customization through personality traits and create personality clusters.

We fit a generalized linear model between the expected value or RMSE and the graph-characteristic covariate vector. In order to obtain population-level estimates of model parameters, we utilize GEE^46^. The multi-user prediction architecture with multiple connected users and the sociobehavioural dataset used for evaluation contains multiple participants that have similar personalities and can be grouped together. If these clusters are ignored in the analysis, they can result in erroneous models^47^. Assuming *P* clusters with $n_p$ observation in $p$th cluster, where $p=1,2 \ldots P$. Let the RMSE for $p$th cluster and $q$th observation be represented by $R_{pq}$ and corresponding $w\times 1$ covariate vector $X_{pq}$, where $w=4$ and $X_{pq}$ is a vector containing degree, closeness, eigenvalue, and pagerank centrality for a given user sample. The response vector for cluster *p* is denoted by $R_p=[R_{p1},R_{p1}, \ldots ,R_{pn_p}]$ with expected value $\mu _p=[ \mu _{p1}, \mu _{p2}, \ldots ,\mu _{pn_p} ]$,

20 $$\begin{aligned} R_p = \mu _p + \epsilon \end{aligned}$$

where $\epsilon$ represents the random error term. We fit a linear model between covariates and the expected value of response vector $\mu _p$^48^,

21 $$\begin{aligned} g(\mu _p) = X_p*\beta \end{aligned}$$

where *g*(.) is known as the link function that depends on the distribution of the response variable and $\beta$ is $w\times 1$ vector containing regression coefficients that need to be estimated. At this point, there are three key design parameters: the identification of groups, the probability distribution of the response variable, and the working correlation structure of the RMSE variable.

#### Group identification

There are two types of nesting involved in the dataset, cross-sectional (across multiple participants) and longitudinal(repeated observations from the same participant over time). While the correlation structure specified in the GEE model accounts for the longitudinal correlation, it is equally important to identify the groups in the nested dataset so the model can account for within-cluster correlation^49^. In order to identify these clusters, we utilize demographic and personality information.

Participants fill out a one-time Big Five personality^50^ survey at the start of the study. The questionnaire response is then processed to obtain a score for the five personality dimensions, extraversion, agreeableness, conscientiousness, openness, and neuroticism on a scale of 1 to 100. In addition to personality traits, we also incorporate gender information.

Based on these six features, the participants are clustered into groups. We use hierarchical clustering that sequentially partitions data and creates a hierarchy of clusters. In order to identify the optimal number of clusters, we build a dendrogram of all observations^51^. Then we cut the tree diagram horizontally such that it captures more than 70% of the data and count the number of clusters above the cutting line.

For this optimal number of clusters, we apply Agglomerative clustering to the data. This clustering method works its way bottom-up starting by treating each object as a cluster, then merging pairs of clusters until the desired number of clusters is reached. For merging clusters, the similarity between sets of observations is quantified by computing a distance metric called linkage between observations across the two pairs of clusters. We utilize ward linkage as it gives the most balanced clusters. We identify 11 optimal clusters with minimum and maximum euclidean distances equal to 46 and 136 respectively. The cluster sizes vary between 20 and 40 points.

#### Distribution of response variable

The link function maps the expected value of the response variable to the linear regression of covariates and is derived from the distribution followed by the response variable. Therefore, we plot the histogram of the RMSE as shown in supplementary information in Fig. 1S. It can be seen that it closely follows a Gaussian distribution. Since the response variable is normal, no transformation is needed and we use the identity link function^52^.

#### Correlation structure

The correlation structure of the response variable accounts for the correlation between different participants within a cluster. For cluster *p* with $n_p$ observations, the working-correlation $\Sigma _p$ is an $n_p \times n_p$ matrix with diagonal entries equal to subject variance and cross-diagonal entries representing the inter-subject correlation. A summary of commonly used working correlation structures is provided in^48^. We conducted multiple experiments with commonly used structures listed in^48^ and observed that the fitted model has the highest confidence in estimated parameters for Autoregressive structure defined by,

22 $$\begin{aligned} \Sigma _p (R_{pi},R_{p(i+m)}) = \alpha ^m \;\;\; for \;\;\; m=0,1,2,...,n_p-j \end{aligned}$$

where the parameter $\alpha$ is estimated from current estimates of $\beta$. For details on the iterative algorithm for estimation of $\alpha$, please refer to^53^. Please note that it is one of the strengths of GEE that even if the chosen correlation structure is not accurate, the model still gives consistent results.

### Ethics approval and consent to participate

The study protocols and informed consent procedure were approved by the Massachusetts Institute of Technology and Partners HealthCare Institutional Review Boards. The study was registered on clinicaltrials.gov (NCT02846077). All participants signed an informed consent form. All methods were performed in accordance with the relevant guidelines and regulations complying with the declaration of helsinki.

## Supplementary Information

Supplementary Information.

## Data Availability

To protect study participants’ privacy and consent and since some of the participants did not consent to share their data with the third-party researchers, raw data will not be publicly available. However, processed features, and graph networks extracted from phone data, which are used to evaluate the models in this work, are available online. The source code to replicate the experiments presented in this work is also available in the same repository. Please find them at https://github.com/comp-well-org/Graph_contagion_emotion_prediction.
