# Supplementary material for: Exploiting social graph networks for emotion prediction
Source: Sci Rep. 2023 Apr 13;13:6069. doi: 10.1038/s41598-023-32825-9 (PMC10100636; doi:10.1038/s41598-023-32825-9)
Supplement: Supplementary file 1 — Supplementary Information. [file 41598_2023_32825_MOESM1_ESM.pdf]

Exploiting Social Graph Networks for Emotion Prediction

Maryam Khalid, Akane Sano

1 Additional Results

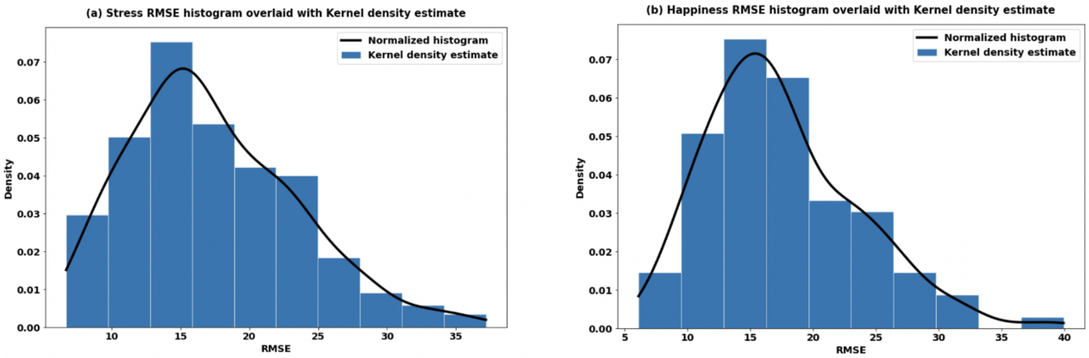

Figure 1: (S) Distribution of the stress and happiness prediction model root mean square error (RMSE). Normalized histogram is shown in blue bars and a kernel density function is fitted to it.

	Average stress score		Standard deviation in stress score	
	Coefficient	P-Value	Coefficient	P_Value
Eigenvalue centrality	-9	0.004	3.5	0.01
Small Degree (D<4)	0.5	0.8	-1.2	0.13
Large Degree (D>4)	-2	0.4	-2	0.17
Closeness Centrality	1.5	0.8	-0.3	0.9
Pagerank Centrality	0.01	0.8	-0.01	0.49

Table 1: (S) GEE results with true happiness score as dependent variable and graph centrality metrics as independent variables.

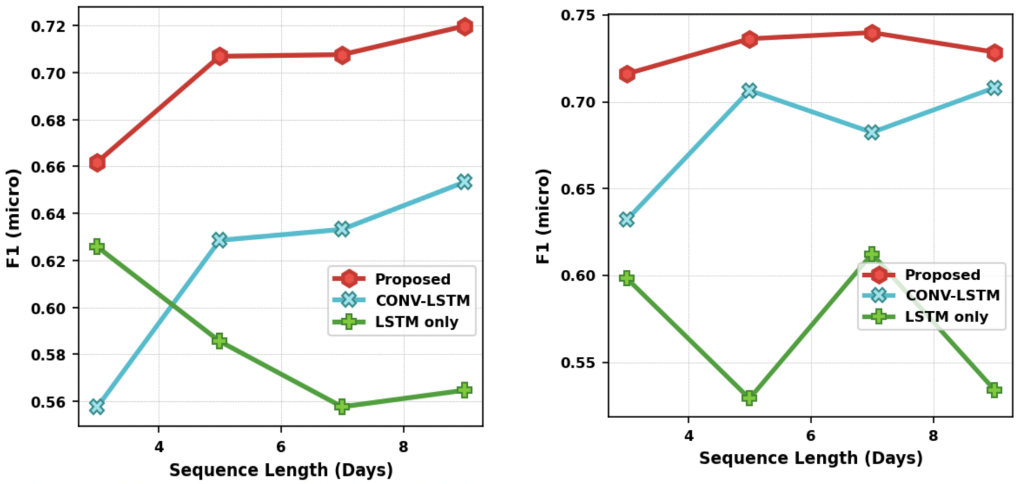

(a) Smaller graph with 10 nodes

(b) Larger graph with 15 nodes

Figure 2: (S) Impact of temporal memory on happiness prediction

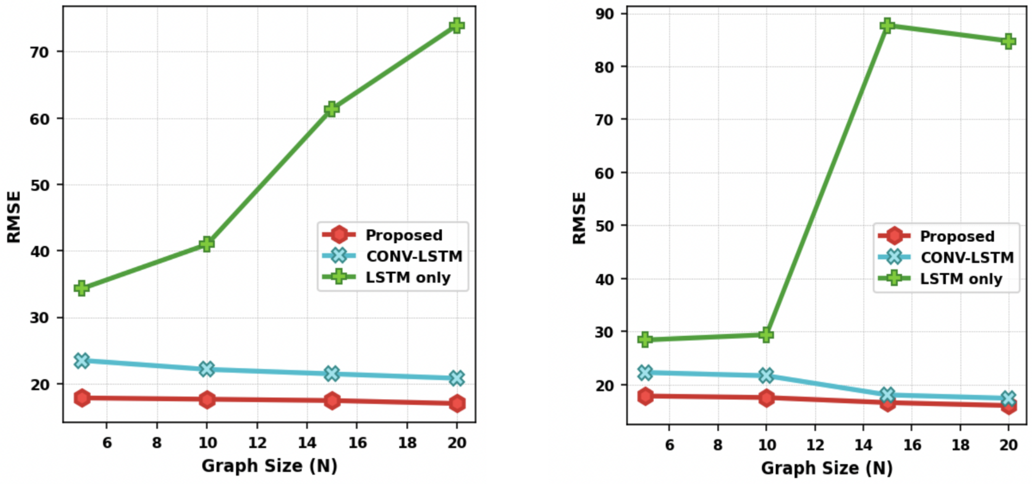

(a) Impact of graph size on stress RMSE

(b) Impact of graph size on happiness RMSE

Figure 3: (S) Impact of graph size on root mean squared error

2 Descriptive Statistics

2.1 Emotion labels distribution

The distribution of self-reported scores for participants across multiple days that they were part of the study is presented through boxplots in Figure. 4 and 5. The x-axis represents a unique user and the corresponding boxplot on the y-axis represents the spread of the corresponding emotion label for that user across multiple days. Each box presents five statistics. The width of the colored box corresponds to the interquartile range(IQR) and the black line inside the box represents the median value. The lower and upper ends of the whiskers show the minimum and maximum values while the black scatter points outside the whisker boundaries correspond to the outliers.

It can be observed from all four plots that there is a huge variation across the median value of emotion scores across users. Not only that, but the IQR also varies a lot. Some participants have a small IQR, meaning their emotion values are not changing a lot on a day-to-day basis. While another large proportion of participants has a large variation in the score. This highlights the dynamic nature of emotion and the need for customized emotion models for each participant. We integrate this customization through rich multi-modal sensor data.

2.2 Network Statistics

The distribution plots of degree and edge strength are presented in Figure.6,7 and 8.

2.3 Phone Data Statistics

The statics of data collected from phones are presented in Table. 2.

**Variable**	**Mean**	**Median**	**25th Percentile**	**75th Percentile**	**Standard deviation**
Daily total call duration (in seconds)	756.42	49	0	507	2750.7
Daily total number of calls	4.4	2	0	5	12.3
Daily total number of SMS	41.7	18	3	50	70.8
Daily screen usage duration (in seconds)	7826.7	6997.8	2994.3	11899.7	41185.7
Daily number of times screen unlocked/used	119	95	45	165	121.2

Table 2: Descriptive statistics for metadata collected from mobile phone

2.4 Mobile sensing data description and statistics

The wearable device worn by the participants on their wrists collected Electrodermal Activity (EDA), Skin Temperature (ST), and 3-axis Accelerometer at 8 GHz. The units of EDA, ST, and Accelerometer are microsiemens (μS), Celsius ($°C$), and gravity (G) respectively. The data for one 24-hr day (0-24) is divided into three 7-hour and one 3-hr windows from 3-10H, 10-17H, 17-24H, and 0-3H. For each window, multiple features from EDA peaks, Skin Conductance Level (SCL), accelerometer, and temperature are computed. For EDA Peak features, the first peaks and artifacts were detected. Then, features listed in Table 3 were computed for both, all detected peaks, and only non-artifact peaks. The statistics for these features for each window (3-10H, 10-17H, 17-24H, and 0-3H) are presented in table 4.

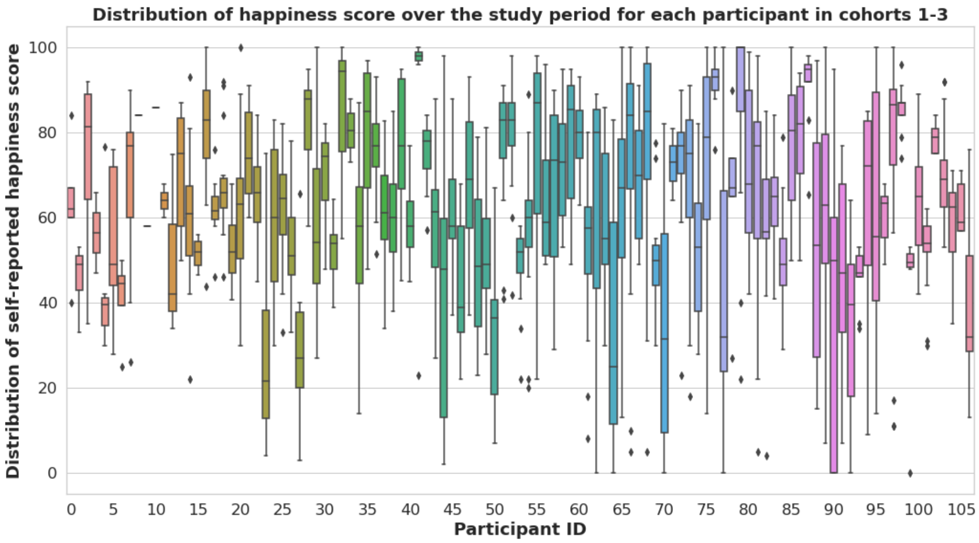

(a) Distribution for participants in cohorts 1,2 and 3

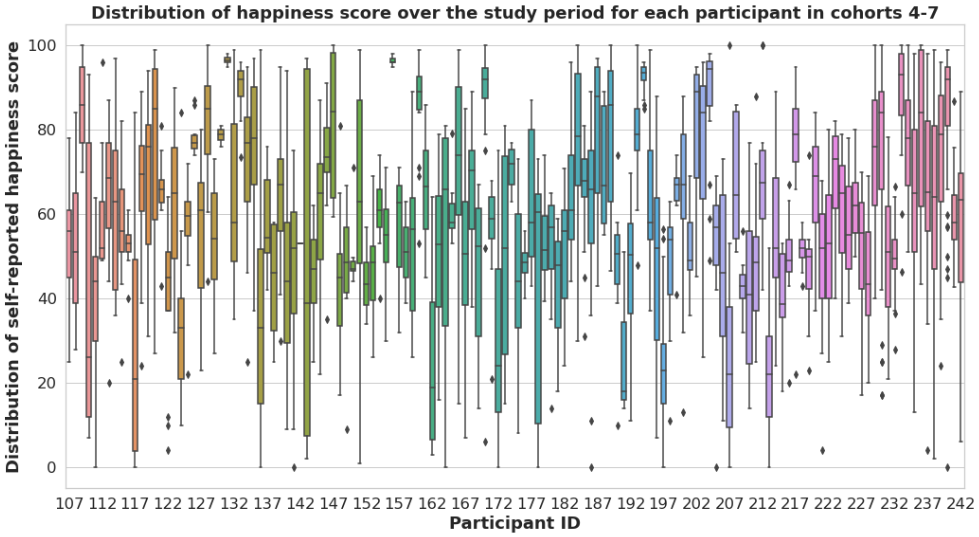

(b) Distribution for participants in cohorts 4,5, 6 and 7

Figure 4: (S) Distribution of self-reported happiness score over the study period for each participant. For better visualization, each participant is assigned a different color.

Furthermore, multiple features about the weather are extracted from DarkSky's weather API [1]. The weather data contained information about temperature, sunlight, Barometric pressure, and the difference between today's weather, and the rolling average and wind. The details of features and their statistics are provided in Table 5 and 6 respectively.

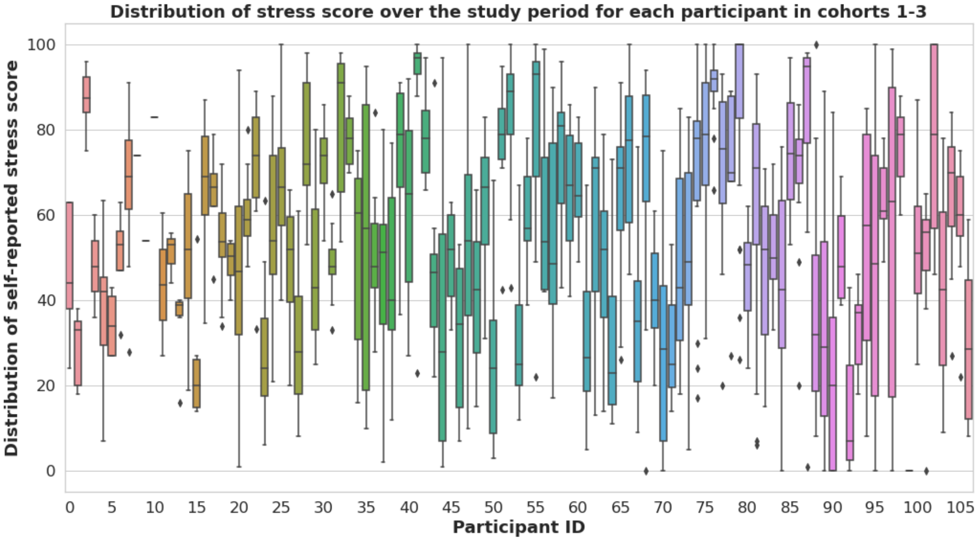

(a) Distribution for participants in cohorts 1,2 and 3

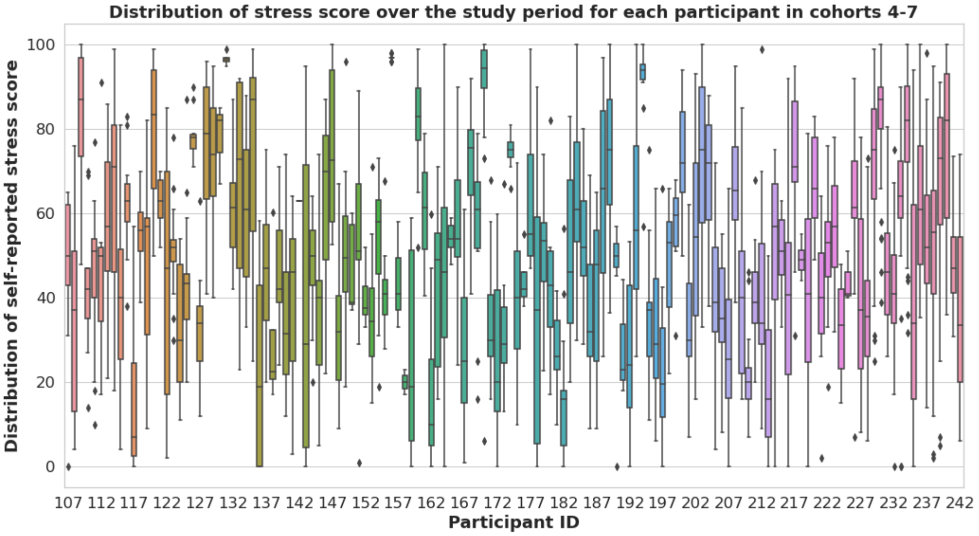

(b) Distribution for participants in cohorts 4,5, 6 and 7

Figure 5: (S) Distribution of self-reported stress score over the study period for each participant

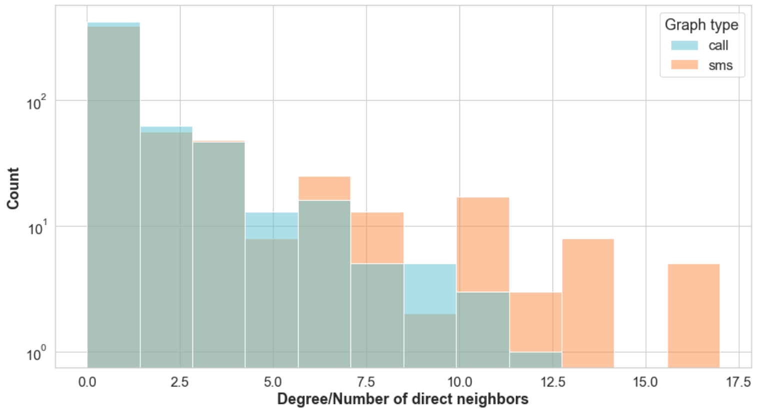

Figure 6: (S) Histogram of the number of direct friends in call and SMS graph networks

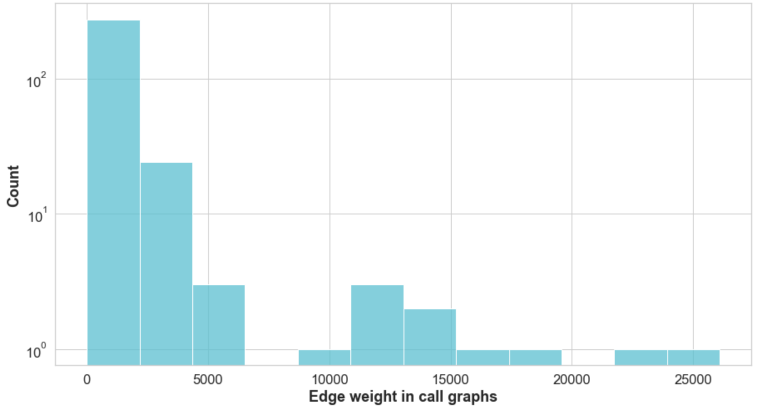

Figure 7: (S) Histogram of the edge strength (duration of calls in seconds during the study period) in call graph networks

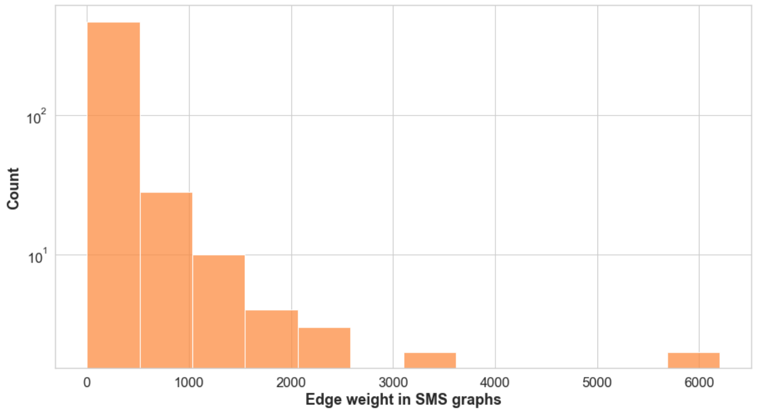

Figure 8: (S) Histogram of the edge strength (weighted sum of the number of SMS exchanged during the study period) in SMS graph networks

**Electrodermal activity (EDA) Peak Features**	
Sum AUC	the sum of the AUC of all peaks for this period where the amplitude of the peak is calculated as the difference from base tonic signal
Sum AUC Full	sum of AUC of peaks where amplitude is calculated as difference from 0
Median RiseTime	median rise time of peaks (seconds)
Median Amplitude	median amplitude of peaks (uS)
Count Peaks	number of peaks detected
SD Peaks 30 min	compute # of peaks per 30 minute epoch, take standard deviation of this signal
Med Peaks 30 min	compute # of peaks per 30 minute epoch, take median of this signal
Percent Med Peak	percentage of signal containing 1 minute epochs with greater than 5 peaks
Percent High Peak	same as percent Med Peak
**Skin Conductance Level (SCL) Features**	
Percent Off	percentage of period where sensor was off
MaxUnnorm	maximum level of un normalized EDA signal
MedUnnorm	median of normalized EDA signal
MeanUnnorm	mean of unnormalized EDA signal
Median Norm	median of z score normalized EDA signal
SD Norm	Standard Deviation of z score normalized EDA signal
Mean Deriv	mean derivative of z score normalized EDA signal (uS/second)
**Accelerometer Features**	
Step Count	number of steps detected
Mean Movement Step Time	average number of samples (at 8Hz) between two steps (aggregated first to 1 minute, then we take the mean of only the parts of this signal occurring during movement)
Stillness Percent	Percentage of time the person spent nearly motionless
Sum Stillness Weighted AUC	weight the peak AUC signal by how still the user was every 5 minutes and sum
Sum Steps Weighted AUC	weight the peak AUC signal by the step count over every 5 minutes and sum
Sum Stillness Weighted Peaks	Multiply the number of peaks every 5 minutes by the amount of stillness during that period
Max Stillness Weighted Peaks	the max value for the # peaks * stillness for any five minute period
Sum Steps Weighted Peaks	divide number of peaks every five minutes by step count and sum
Med Steps Weighted Peaks	average value for the number of peaks / step count every 5 mins
**Skin Temperature(ST) Features**	
Max Raw Temp	the maximum of the raw temperature signal (°C)
Min Raw Temp	the minimum of the raw temperature signal (°C)
SD Raw Temp	the standard deviation of the raw temperature signal
Med Raw Temp	median of the same
Sum Temp Weighted AUC	sum of peak AUC divided by the average temp every 5 mins
Sum Temp Weighted Peaks	number of peaks divided by the average temp every 5 mins
Max Temp Weighted Peaks	the maximum number of peaks in any 5 minute period divided by the average temp
SD Stillness Temp	std dev of the temperature recorded during periods when the person was still
Med Stillness Temp	median of the temperature when the person was still

Table 3: Description of features extracted from wearable device

**Feature**	**Mean**	**Median**	**25th Percentile**	**75th Percentile**	**Standard deviation**
0H-3H:sumAUC	58.96	8.07	1.27	39.98	147.19
0H-3H:sumAUCFull	342.93	16.93	1.59	126.1	1036.55
0H-3H:countPeaks	34.15	7	2	27	71.28
0H-3H:sdPeaks30min	6.35	1.37	0.75	5.64	11.92
0H-3H:medPeaks30min	3.31	0.5	0	2	9.64
0H-3H:percentMedPeak	0.84	0	0	0	2.69
0H-3H:percentHighPeak	0.84	0	0	0	2.69
0H-3H:sumAUCNo Artifact	33.37	5.92	0.5	25.21	73.81
0H-3H:sumAUC FullNoArtifact	216.88	11.69	0.93	75.45	624.42
0H-3H:countPeaks NoArtifact	18.84	4	1	16	38.02
0H-3H:sdPeaks30 minNoArtifact	3.63	0.76	0.37	3.09	7.11
0H-3H:medPeaks30 minNoArtifact	1.7	0	0	1	4.63
0H-3H:percentMed PeakNoArtifact	0.19	0	0	0	0.78
0H-3H:percentHigh PeakNoArtifact	0.19	0	0	0	0.78
0H-3H:sclPercentOff	12.12	0	0	14.45	23.75
0H-3H:sclMaxUnnorm	1.05	0.61	0.27	1.35	1.24
0H-3H:sclMedUnnorm	0.32	0.21	0.07	0.44	0.4
0H-3H:sclMeanUnnorm	0.37	0.25	0.09	0.51	0.43
0H-3H:sclMedianNorm	0.14	0.09	-0.19	0.4	0.48
0H-3H:sclSDnorm	0.36	0.22	0.1	0.48	0.38
0H-3H:sclMeanDeriv	0	0	0	0	0
0H-3H:stepCount	575.58	380	142.5	768.8	636.92
0H-3H:stillnessPercent	0.73	0.76	0.59	0.91	0.21
0H-3H:sumStillness WeightedAUC	26.66	3.79	0.41	17.93	65.15
0H-3H:sumSteps WeightedAUC	16.97	1.23	0.12	7.95	48.45
0H-3H:sumStillness WeightedPeaks	16.23	3.6	1.4	12	34.38
0H-3H:maxStillness WeightedPeaks	4.17	2	0.8	4	6.46
0H-3H:sumSteps WeightedPeaks	10.41	1.14	0.33	5.52	27.48
0H-3H:medSteps WeightedPeaks	0.02	0	0	0	0.12
0H-3H:sumTemp WeightedAUC	-20.76	-1.22	-10.91	0	65.87
0H-3H:sumTemp WeightedPeaks	-10	-0.68	-6.41	0.91	34.1

**Feature**	**Mean**	**Median**	**25th Percentile**	**75th Percentile**	**Standard deviation**
0H-3H:maxTemp WeightedPeaks	2.29	0.07	0	2.62	4.45
0H-3H:maxRawTemp	35.32	35.44	33.9	36.9	2.28
0H-3H:minRawTemp	29.29	29.7	27.6	31.6	3.16
0H-3H:sdRawTemp	1.35	1.24	0.82	1.72	0.73
0H-3H:medRawTemp	32.65	32.88	31.4	34.1	2.09
0H-3H:sd StillnessTemp	1.22	1.13	0.73	1.55	0.66
0H-3H:med StillnessTemp	32.94	33.1	31.62	34.3	2.1
3H-10H:sumAUC	113.76	25.41	5.36	109.07	216.26
3H-10H:sumAUCFull	819.19	69.37	12.41	515.77	1942.99
3H-10H:countPeaks	67.13	20	6	76	110.94
3H-10H:sd Peaks30min	7.62	2.2	0.8	9.03	11.65
3H-10H:med Peaks30min	1.52	0	0	1	3.75
3H-10H:percent MedPeak	0.7	0	0	0.48	1.72
3H-10H:percent HighPeak	0.7	0	0	0.48	1.72
3H-10H:sumAUC NoArtifact	82.66	16.83	3.78	69.55	168.01
3H-10H:sumAUC FullNoArtifact	681.46	45.78	8.6	357.79	1707.21
3H-10H:count PeaksNoArtifact	47.93	11	3	42.9	90.56
3H-10H:sdPeaks30 minNoArtifact	5.69	1.25	0.48	5.51	10.02
3H-10H:medPeaks30 minNoArtifact	0.88	0	0	1	2.52
3H-10H:percentMed PeakNoArtifact	0.41	0	0	0	1.28
3H-10H:percentHigh PeakNoArtifact	0.41	0	0	0	1.28
3H-10H:sclPercentOff	11.69	0	0	11.47	22.56
3H-10H:scl MaxUnnorm	1.55	1.08	0.55	2	1.56
3H-10H:scl MedUnnorm	0.38	0.3	0.14	0.54	0.37
3H-10H:scl MeanUnnorm	0.45	0.36	0.19	0.63	0.41
3H-10H:scl MedianNorm	0.26	0.27	-0.01	0.54	0.41
3H-10H:sclSDnorm	0.49	0.4	0.2	0.7	0.38
3H-10H:sclMeanDeriv	0	0	0	0	0
3H-10H:stepCount	581.22	388	127	839.5	603.91

**Feature**	**Mean**	**Median**	**25th Percentile**	**75th Percentile**	**Standard deviation**
3H-10H:mean MovementStepTime	876.28	485.01	250.78	990.57	1168.21
3H-10H:stillness Percent	0.9	0.92	0.86	0.97	0.08
3H-10H:sum StillnessWeightedAUC	89.47	16.46	3.44	71.39	187.42
3H-10H:sum StepsWeightedAUC	71.26	8.83	1.47	50.37	161.09
3H-10H:sum StillnessWeightedPeaks	52.68	13.2	3.8	48.9	98.02
3H-10H:max StillnessWeightedPeaks	7.52	3	1.6	9	9.76
3H-10H:sum StepsWeightedPeaks	41.83	6.67	1.49	31.56	86.42
3H-10H:med StepsWeightedPeaks	0.02	0	0	0	0.14
3H-10H:sum TempWeightedAUC	-62.85	-8.53	-47.08	-0.59	153.22
3H-10H:sum TempWeightedPeaks	-33.68	-5.07	-28.76	0	78.8
3H-10H:max TempWeightedPeaks	2.99	0.38	0	3.64	5.6
3H-10H:max RawTemp	37.02	37.2	36	38.1	1.73
3H-10H:min RawTemp	29.05	29.5	27.38	31.4	3.25
3H-10H:sd RawTemp	1.63	1.55	1.2	1.96	0.61
3H-10H:med RawTemp	33.95	34.1	32.9	35.2	1.77
3H-10H:sd StillnessTemp	1.45	1.39	1.1	1.72	0.51
3H-10H:med StillnessTemp	34.14	34.3	33.1	35.4	1.74
10H-17H:sum AUC	149.92	37.05	6.17	150.23	293.19
10H-17H:sum AUCFull	870.43	105.47	12.51	635.51	2158.79
10H-17H:median RiseTime	1.93	1.92	1.88	2	0.21
10H-17H:median Amplitude	0.2	0.19	0.16	0.23	0.08
10H-17H:countPeaks	85.88	30	8	102	134.41
10H-17H:sd Peaks30min	10.16	3.47	1.03	12.96	15.22
10H-17H:med Peaks30min	1.81	0	0	1.5	4.44

Feature	Mean	Median	25th Percentile	75th Percentile	Standard deviation
10H-17H:percent MedPeak	1.07	0	0	0.95	2.27
10H-17H:percent HighPeak	1.07	0	0	0.95	2.27
10H-17H:sum AUCNoArtifact	58.79	19.75	3.63	69.39	95.58
10H-17H:sum AUCFullNoArtifact	370.7	54.81	7.19	319.68	817.64
10H-17H:count PeaksNoArtifact	34.88	15	3	44	49.77
10H-17H:sd Peaks30minNoArtifact	3.81	1.68	0.52	5.17	5.09
10H-17H:med Peaks30minNoArtifact	0.88	0	0	1	2.03
10H-17H:percent MedPeakNoArtifact	0.09	0	0	0	0.31
10H-17H:percent HighPeakNoArtifact	0.09	0	0	0	0.31
10H-17H:sclPercentOff	12.45	2.3	0	14.24	22.08
10H-17H:scl MaxUnnorm	1.72	1.08	0.45	2.29	1.95
10H-17H:scl MedUnnorm	0.24	0.16	0.05	0.34	0.3
10H-17H:scl MeanUnnorm	0.32	0.22	0.09	0.45	0.35
10H-17H:scl MedianNorm	-0.02	-0.05	-0.26	0.19	0.35
10H-17H:sclSDnorm	0.48	0.37	0.18	0.69	0.4
10H-17H:scl MeanDeriv	0	0	0	0	0
10H-17H:stepCount	3040.2	2831	1768.7	4097	1805.32
10H-17H:mean MovementStepTime	253.45	218.13	160.57	302.54	143.27
10H-17H:stillness Percent	0.6	0.6	0.48	0.71	0.18
10H-17H:sum StillnessWeightedAUC	33.98	9.13	1.67	35.57	63.48
10H-17H:sum StepsWeightedAUC	15.31	3.1	0.47	12.91	33.66
10H-17H:sum StillnessWeightedPeaks	21.66	8.4	2.6	25.8	33.26
10H-17H:max StillnessWeightedPeaks	5.13	2.4	1.2	7	6.22
10H-17H:sum StepsWeightedPeaks	9.9	2.85	0.7	9.35	18.97
10H-17H:med StepsWeightedPeaks	0	0	0	0	0.02

Feature	Mean	Median	25th Percentile	75th Percentile	Standard deviation
10H-17H:sum TempWeightedAUC	-10.47	-0.09	-13	4.36	121.89
10H-17H:sum TempWeightedPeaks	-3.27	0	-7.96	8.88	58.33
10H-17H:max TempWeightedPeaks	6.63	2.7	0.29	7.57	10.98
10H-17H:max RawTemp	35.5	35.4	34.1	37	2.33
10H-17H:min RawTemp	26.22	26.6	24.3	28.5	3.28
10H-17H:sd RawTemp	1.77	1.66	1.27	2.17	0.68
10H-17H:med RawTemp	31.54	31.6	30.4	32.6	1.75
10H-17H:sd StillnessTemp	1.55	1.44	1.11	1.9	0.63
10H-17H:med StillnessTemp	31.94	32	30.8	33.22	1.84
17H+:sumAUC	205.12	34.88	5.34	180.86	457.92
17H+:sumAUCFull	1276.89	93.33	10.18	812.79	3370.56
17H+:countPeaks	107.94	29	7	120	187.48
17H+:sdPeaks 30min	11.99	3.05	0.84	13.84	19.65
17H+:med Peaks30min	2.21	0	0	1	6.35
17H+:percent MedPeak	1.48	0	0	1.19	3.36
17H+:percent HighPeak	1.48	0	0	1.19	3.36
17H+:sum AUCNoArtifact	71.45	19.32	2.97	80.46	124.16
17H+:sum AUCFullNoArtifact	477.88	50.83	5.8	344.79	1102.34
17H+:count PeaksNoArtifact	40.38	14	3	50.9	61.92
17H+:sd Peaks30minNoArtifact	4.16	1.6	0.42	5.41	5.9
17H+:med Peaks30minNoArtifact	1.04	0	0	1	2.65
17H+:percent MedPeakNoArtifact	0.1	0	0	0	0.35
17H+:percent HighPeakNoArtifact	0.1	0	0	0	0.35
17H+:sclPercentOff	11.7	0	0	14.58	21.15
17H+:sclMaxUnnorm	1.8	0.98	0.36	2.31	2.21
17H+:sclMedUnnorm	0.25	0.15	0.04	0.33	0.32
17H+:sclMeanUnnorm	0.34	0.22	0.08	0.46	0.4

Feature	Mean	Median	25th Percentile	75th Percentile	Standard deviation
17H+:sclMedianNorm	-0.01	-0.05	-0.27	0.21	0.38
17H+:sclSDnorm	0.49	0.34	0.15	0.72	0.45
17H+:sclMeanDeriv	0	0	0	0	0
17H+:stepCount	3082.07	2711	1646	4082	1977.16
17H+:mean MovementStepTime	264.79	229.02	165.32	317.35	161.34
17H+:stillnessPercent	0.54	0.53	0.4	0.67	0.19
17H+:sum StillnessWeightedAUC	33.78	7.18	1.14	31.48	70.11
17H+:sum StepsWeightedAUC	15.1	2.33	0.29	11.59	36.6
17H+:sum StillnessWeightedPeaks	20.61	6.61	2.01	22.22	35.2
17H+:max StillnessWeightedPeaks	4.69	2.14	1	6.13	5.99
17H+:sum StepsWeightedPeaks	9.23	2.17	0.46	8.22	19.22
17H+:med StepsWeightedPeaks	0.01	0	0	0	0.03
17H+:sum TempWeightedAUC	-19.96	0	-12.27	3.61	194.27
17H+:sum TempWeightedPeaks	-7.71	0	-7.66	7	82.94
17H+:maxTemp WeightedPeaks	6.28	2.21	0.15	7.05	10.88
17H+:maxRawTemp	34.77	34.7	33.3	36	2.34
17H+:minRawTemp	25.61	26	23.7	28.3	3.65
17H+:sdRawTemp	1.73	1.59	1.19	2.17	0.76
17H+:medRawTemp	31.17	31.2	29.9	32.4	1.82
17H+:sdStillnessTemp	1.46	1.33	0.99	1.79	0.67
17H+:medStillnessTemp	31.51	31.6	30.4	32.9	1.92

Table 4: Descriptive statistics for physiological features extracted from the wearable device. The feature name is preceded by the time window over which the feature value was extracted.

**Weather Features**	
Sunrise	Sunrise time UTC
Moon_phase	The moon phase value on a scale of $0 - 1$(new moon-full moon)
Apparent_temp_max	Maximum apparent temperature of the day in Fahrenheit
Apparent_temp_min	Minimum apparent temperature of the day in Fahrenheit
Temperature_max	Maximum temperature of the day in Fahrenheit
Temperature_min	Minimum temperature of the day in Fahrenheit
avg_cloud_cover	Percentage of sky covered by cloud on a sclae of 0-1
avg_dew_point	Average dew point temperature
avg_humidity	Daily average value of humidity on a scale of 0-1
avg_pressure	Average atmospheric pressure on the sea level in hPa
Morning_pressure_change	Trinary value of pressure difference between midnight and noon (rising, falling, steady)
Evening_pressure_change	Trinary value of pressure difference between noon and midnight (rising, falling, steady)
avg_visibility	Average visibility in meters
weather_precip_probability	Precipitation probability
Temperature_rolling_mean	Rolling average of temperature
Temperature_rolling_std	Rolling standard deviation in temperature
Temperature_today_vs_avg_past	Difference between today's temperature and rolling average
apparentTemperature_rolling_mean	Rolling average of apparent temperature
apparentTemperature_rolling_std	Rolling standard deviation in apparent temperature
apparentTemperature_today_vs_avg_past	Difference in today's apparent temperature and its rolling average
pressure_rolling_mean	Rolling average of pressure
pressure_rolling_std	Rolling standard deviation of pressure
pressure_today_vs_avg_past	Difference between today's pressure and its rolling average
cloudCover_rolling_mean	Rolling average of cloud cover
cloudCover_rolling_std	Rolling standard deviation in cloud cover
cloudCover_today_vs_avg_past	Difference between today's cloud cover and its rolling average
humidity_rolling_mean	Rolling average of humidity
humidity_rolling_std	Rolling standard deviation in humidity
humidity_today_vs_avg_past	Difference between today's humidity and its rolling average
windSpeed_rolling_mean	Rolling average of wind speed
windSpeed_rolling_std	Rolling standard deviation in wind speed
windSpeed_today_vs_avg_past	Difference between today's wind speed and its rolling average
precipProbability_rolling_mean	Rolling average of precipitation probability
precipProbability_rolling_std	Rolling standard deviation in precipitation probability
precipProbability_today_vs_avg_past	Difference between current precipitation probability and its rolling average
sunlight	Duration of sunlight in seconds
quality_of_day	Quality of the day define in terms of 8 categories in the range $\{-4, 4\}$: clear= 4, partly-cloudy= 3, cloudy= 2, wind=1, fog= -1, rain= -2, sleet= -3, snow= -4
avg_quality_of_day	Average value for quality_of_day
precipType	Type of precipitation as integer: None = 0, Rain = 1, Hail = 2, Sleet = 3, Snow = 4, Other = 5
max_precip_intensity	Maximum Precipitation volume in mm
median_wind_speed	Median wind speed of the day in meter/sec
median_wind_bearing	Median wind bearing of the day in degrees

Table 5: Description of features extracted from weather API

Feature	Mean	Median	25th Percentile	75th Percentile	Standard Deviation
weather_sunrise	23743.66	23800	22788	25061	1596.66
weather_moon_ phase	0.51	0.53	0.28	0.75	0.28
weather_apparent_ temp_max	44.24	44.84	26.66	60.35	19.71
weather_apparent _temp_min	25.58	26.18	10.27	42.15	20.47
weather_ temperature_max	47.69	47.9	34.16	60.35	16.1
weather_ temperature_min	32.4	32.91	19.84	45.43	15.76
weather_avg_ cloud_cover	0.37	0.26	0.17	0.5	0.28
weather_avg_dew_point	27.18	28.01	12.78	41.05	18.06
weather_avg_humidity	0.63	0.61	0.5	0.73	0.15
weather_avg_pressure	1015.85	1016.23	1010.24	1021.42	9.07
weather_morning_ pressure_change	0.16	1	-1	1	0.91
weather_evening_ pressure_change	0.04	0	-1	1	0.94
weather_avg_visibility	9.1	9.73	8.92	10	1.4
weather_ precip_probability	0.25	0	0	0.6	0.33
weather_temperature_ rolling_mean	39.91	39.99	26.29	52.41	14.26
weather_temperature_ rolling_std	7.23	6.98	5.54	8.57	2.35
weather_temperature_ today_vs_avg_past	0.14	0.38	-5.38	5.94	8.73
weather_apparent Temperature_rolling_mean	35.01	34.79	19.5	51.56	17.99
weather_apparent Temperature_rolling_std	8.94	8.45	6.8	11.05	2.94
weather_apparent Temperature_today_ vs_avg_past	0.12	0.33	-6.96	7.54	10.85
weather_pressure_ rolling_mean	1015.55	1015.96	1010.68	1020.79	6.83
weather_pressure_ rolling_std	6.18	5.72	3.8	7.86	2.99
weather_pressure_ today_vs_avg_past	0.29	0.61	-5.49	6.62	9.61
weather_cloudCover_ rolling_mean	0.37	0.32	0.22	0.47	0.18
weather_cloudCover_ rolling_std	0.22	0.17	0.1	0.33	0.14

Feature	Mean	Median	25th Percentile	75th Percentile	Standard Deviation
weather_cloudCover_ today_vs_avg_past	0	-0.02	-0.12	0.12	0.22
weather_humidity_ rolling_mean	0.64	0.63	0.54	0.72	0.12
weather_humidity_ rolling_std	0.16	0.17	0.13	0.19	0.04
weather_humidity_ today_vs_avg_past	-0.01	-0.01	-0.13	0.11	0.16
weather_windSpeed_ rolling_mean	8.91	9.56	7.57	11.11	3.65
weather_windSpeed_ rolling_std	3.49	3.35	2.42	4.4	1.49
weather_windSpeed_ today_vs_avg_past	-0.04	-0.34	-1.75	1.55	3.47
weather_precipProbability _rolling_mean	0.06	0.03	0	0.09	0.09
weather_precipProbability _rolling_std	0.13	0.11	0.01	0.21	0.11
weather_precipProbability _today_vs_avg_past	-0.01	-0.01	-0.06	0.03	0.13
weather_sunlight	40908.49	40127	38595	41641	3726.93
weather_quality_of_day	1.49	3	-2	4	2.83
weather_precipType	0.88	0	0	1	1.37
weather_avg_ quality_of_day	3.02	3.52	2.54	3.75	1.1
weather_max_ precip_intensity	0.02	0	0	0.02	0.04
weather_median_ wind_speed	8.8	8.78	6.3	11.23	4.36
weather_median_ wind_bearing	240.78	267	209	300	89.61

Table 6: Descriptive statistics for weather features obtained from Darksky API.

3 Graph Convolutional Networks

In this section, we provide a brief derivation of GCN forward propagation rule which incorporated the graph degree and laplacian matrix. The normalized graph Laplacian L is a symmetric matrix defined as,

$$L = I_N - D^{-1/2} A D^{-1/2}$$

where I_N is $N \times N$ identity matrix. The spectral convolution on a graph is simply fourier domain multiplication of node feature signal x (assume scalar for ease of understanding) with a filter h_θ parametrized by θ[2],

$$h(\theta) * x = U h_\theta(\Lambda) U^T x \tag{1}$$

where U and Λ are the eigenvector and eigenvalue matrix of L: $L = U \Lambda U^T$. To avoid the expensive computational cost of eigenvalue decomposition of L, a truncated Chebychev polynomial expansion

of $h_\theta(\Lambda)$ is utilized to obtain the following simplified expression[3],

$$h(\theta) * x = L\theta x \tag{2}$$

The details are skipped in the interest of space. For the detailed derivation, please refer to [2][3]. The expression in eq. (2) can be generalized for any signal $X \in \mathbb{R}^{NxC}$ for graph size N and channels C,

$$h(\theta) * x = \tilde{D}^{-1/2}\tilde{A}\tilde{D}^{-1/2}X\Theta \tag{3}$$

where $\tilde{A} = A + I_N$ is the adjacency matrix with added self-loops and $\tilde{D_{ii}} = \sum_j \tilde{A}_{ij}$. Θ represents the network parameters to be learned.

References

[1] "Dark sky forecast api [online]." https://developer.forecast.io/, 2016.

[2] T. Kipf and M. Welling, "Semi-supervised classification with graph convolutional networks," *ArXiv*, vol. abs/1609.02907, 2017.

[3] D. K. Hammond, P. Vandergheynst, and R. Gribonval, "Wavelets on graphs via spectral graph theory," *Applied and Computational Harmonic Analysis*, vol. 30, no. 2, pp. 129–150, 2011.
